# Protective effects of *Euphorbia heterophylla* against testicular degeneration in streptozotocin-induced diabetic rats in relation to phytochemical profile

**DOI:** 10.1371/journal.pone.0314781

**Published:** 2025-01-16

**Authors:** Ahmed M. Nagy, Heba A. Fahmy, Mohamed F. Abdel-Hameed, Rehab F. Taher, Alaa M. Ali, Mohamed M. Amin, Sherif M. Afifi, Tuba Esatbeyoglu, Mohamed A. Farag, Abdelsamed I. Elshamy

**Affiliations:** 1 Department of Animal Reproduction & AI, National Research Center, Giza, Egypt; 2 Pharmacognosy Department, Faculty of Pharmacy, Modern University for Technology & Information, Cairo, Egypt; 3 Department of Pharmacology, Research Centre, Giza, Egypt; 4 Department of Natural Compounds Chemistry, National Research Centre, Giza, Egypt; 5 Department of Pathology, Faculty of Veterinary Medicine, Cairo University, Giza, Egypt; 6 Department for Life Quality Studies, Rimini Campus, University of Bologna, Rimini, Italy; 7 Department of Molecular Food Chemistry and Food Development, Institute of Food and One Health, Gottfried Wilhelm Leibniz University Hannover, Hannover, Germany; 8 Pharmacognosy Department, Faculty of Pharmacy, Cairo University, Cairo, Egypt; Helwan University, EGYPT

## Abstract

**Background:**

Diabetes mellitus (DM) poses a major risk to human health due to an array of implications, one of which is a detrimental effect on the testicular and reproductive functions. *Euphorbia heterophylla* is widely recognized for its medicinal properties worldwide.

**Methods and findings:**

The objective of this study was to profile *E*. *heterophylla* ethanol extract (EH-EtOH) and elucidate its protective role in oxidative stress, relieving inflammatory action of hyperglycemia-induced testicular degeneration and restoring the normal histological structure with physiological properties of testicular tissue in streptozotocin (STZ)-induced DM. High-resolution ultra-performance liquid chromatography-mass spectrometry (UHPLC-ESI-Orbitrap-MS) analysis was employed to analyze the compounds present in EH-EtOH. The protective effect of EH-EtOH against testicular degeneration in the rat model of DM was evaluated by measuring improvements in blood glucose levels, body weight, testicular inflammation, oxidative damage, testicular microcirculation impairment, and apoptosis of testicular cells induced by STZ. The chemical profiling of EH-EtOH revealed the presence of 52 compounds, including phenolic acids, flavonoids, coumarins, phloroglucinols, and triterpenes. Notably, this study identified isovitexin-*C*-hexoside, isorhamnetin-*O*-hexoside, diosmetin, and halfordin for the first time in Euphorbia species. Treatment with EH-EtOH effectively mitigated the damage caused by STZ, as evidenced by restored testosterone (T4) levels and antioxidant capacity, reduced expression of pro-inflammatory cytokines, improved testicular microcirculation, and inhibition of apoptosis in the testes.

**Conclusions:**

These results emphasize the potential therapeutic effect of *E*. *heterophylla* on DM related to male infertility and reproductive dysfunctions via its antioxidant/angiogenetic /anti-apoptotic effect.

## 1. Introduction

Diabetes mellitus (DM) is a chronic metabolic disorder manifested by an increase in glucose level attributed to insulin insufficiency as a result of the pancreatic defect [1]. The high prevalence of DM is considered a major threat to human health, and to further negatively affect the structure and function of numerous organs including the liver, kidney, heart, brain, and retina in addition to the testis [2, 3]. Previous studies on animals and humans revealed that hyperglycemia affects the testicular and reproductive functions as manifested by reduced T4 level, and failures of all sperm parameters including sperm count, sperm motility, spermatogenesis, and seminal fluid volume [4, 5]. Consequently, the protection of the reproductive system in DM patients seems warranted especially considering that action mechanisms of diabetes-induced male reproductive dysfunction are not fully understood. It is believed that hyperglycemia-induced oxidative stress, inflammation and hormonal imbalance are the main causes of testicular damage [6].

It is likewise known that DM could increase cytokines´ levels like tumor necrosis factor-*α* (TNF-*α*), cyclooxygenase 2 (COX-2) and Prostaglandin E2 (PGE2) leading to systemic inflammation and subsequently insulin resistance and alterations of insulin signaling [7, 8]. Interleukin 1β (IL-1β), is one of the important inflammatory markers associated with type-2 DM which in turn modifies β-cell apoptosis causing its damage subsequently affects insulin secretion [7], and suggestive that anti-inflammatory drugs can aid in diabetes management [8].

Oxidative stress is caused by an imbalance between the production of reactive oxygen species (ROS) and the antioxidant defense system. ROS can cause DNA damage, lipid peroxidation and protein oxidation, leading to cellular death [9]. Developing strategies to protect male reproductive health from diabetes-induced damage using antioxidants seems as potential therapeutic drug management for diabetic patients.

The testicular microvascular supply shortage related to hyperglycemia remains the obvious mechanism correlated to the testicular cell apoptosis and destruction where the decreased vascular endothelial growth factor (VEGF) expression and impaired angiogenesis caused by chronic hyperglycemia could lead to inadequate blood supply to the testicular tissue [10], resulting in hypoxia and increased oxidative stress which in turn trigger apoptosis of testicular cells and ultimately lead to testicular destruction [11].

Natural products have been well characterized as a potential source of pharmaceuticals, with more than 50% of all pharmaceuticals derived from natural products [12]. With 300 genera, over 7,500 species, and widespread distribution across numerous habitats, Euphorbiaceae family represents one of the largest plant families [13]. Plenty of *Euphorbia* plants have been utilized in traditional medicine for treating several diseases including, gonorrhoea, warts, migraines, intestinal parasites, and skin-related disorders. Additionally, an array of *Euphorbia* metabolites, including diterpenes and phenolics have been well reported for their antibacterial, anticancer, PGE2-inhibitory, anti-multidrug-resistant, prolyl endopeptidase inhibitory, antifeedant, anti-HIV, and analgesic, antidiabetic and antioxidant effects [14–18]. In traditional medicine, *E*. *heterophylla* leaf has been utilized as a laxative, antigonorrheal, a migraine remedy, and a wart cure. The leaves are utilized as a cough treatment and anticonvulsant in some areas of Kogi State, Nigeria. According to published research, *E*. *heterophylla* is a highly rich source of flavonoids, phenolic acid [19], terpenoids [20], alkaloids, saponins, tannins [21], and essential oils [13].

This study was planned to: (i) perform a thorough metabolites profile analysis of the ethanolic extract of *E*. *heterophylla* aerial part (EH-EtOH) using UHPLC-ESI-Orbitrap-MSUHPLC-ESI-Orbital Trap-MS; (ii) investigate the effectiveness of EH-EtOH to mitigate testicular degeneration in diabetic rats; and (iii) determine the action mechanisms associated with the metabolites profile employing biochemical and histopathological assays.

## 2. Materials and methods

### 2.1. Plant material collection, authentication

The whole plant of *E*. *heterophylla* was collected from the delta of the Nile region of Egypt, specifically the El Menoufia governorate (30°20’49"N 30°52’14"E) during the flowering period in May 2021. Authentication of the plant material was carried out by Professor of Plant Ecology Ahmed M. Abdel Gawad, with voucher number EHxYA-677-zRH/21-04377 submitted at the Faculty of Science Herbarium, Mansoura University, Egypt.

### 2.2. Extraction procedures for chemical analysis and bioassays

The collected plant material was cleaned of all dirt and sand, and then placed into an open, shaded chamber that was completely dry and allowed to dry at room temperature. A sanitized plant grinder was used to grind the plant material into powder once it had completely dried. 675 g of powdered plant material was extracted using maceration in 70% EtOH (6 L X 3) for one week at room temperature 25–28 °C, and then filtered. Collected extracts were obtained after filtration and dried entirely at low pressure to produce 28.7 g of dark black gummy. After that, the extract which was free of any EtOH was refrigerated at 4 °C until it was subjected to chemical and biological analysis.

### 2.3. Analysis of the metabolites profiling using UHPLC-ESI-Orbital Trap-MS

The metabolites profiling of the ethanolic extract of *E*. *heterophylla* (EH-EtOH) was performed using the ultra-performance liquid chromatography-mass spectrometry analysis (UHPLC-ESI-Orbital Trap-MS) according to a previous protocol [22]. A 500 mg C18 cartridge preconditioned with 100% methanol followed by milli-Q water. 1 mL of Aliquoted sample for UPLC-MS analysis were placed on the cartridge, and further eluted three times with 0.5 mL of methanol. The eluent was then evaporated under a nitrogen stream, and the dry residue left behind was re-suspended in 0.5 mL of 100% methanol. Then, using UPLC-MS in both negative and positive modes and triplicates (n = 3), 2 μL of the suspended fluid was injected on UPLC-MS. The exact settings stated were used to perform chromatographic separations on an Acquity UPLC system Waters, Milford, MA, USA. The system was outfitted with an HSS T3 column (100 ×1.0 mm, particle size 1.8 μm), applying the same parameters as previously reported [23]

### 2.4. Biological assays

#### 2.4.1. Drugs and chemicals

Streptozotocin (STZ) was purchased from Sigma-Aldrich Chemical Co. (St. Louis, MO, USA) CAS#:18883-66-4, Ethanol (96%) was purchased from Merck Millipore (St. Louis, MO, USA) CAS #: 64-17-5). Catalase (cat# CA 25 17) and GSH (cat# GR 25 11) colorimetric kits were purchased from BioDiagnostic (Giza, Egypt). (PGE2, cat# SL0601Ra), COX-2 (cat# SL0218Ra), UGT-glucoronosyl transferase (cat# SL1171Ra), IL-1*β* (cat# SL0402Ra) and TNF-*α*(cat# SL0722Ra), and VEGF (, cat# SL0740Ra) and Estrogen (E2, cat# SL0271Ra) ELISA kits were obtained from SunLong Biotech Co., LTD (Hangzhou, China). While T4 (cat# E-EL-0155) was obtained from Elabscience^®^, Texas, USA.

#### 2.4.2. Determination of acute toxicity

According to the procedures outlined by [24], this study utilized 2-month-old male Swiss albino mice weighing an average of 20.4 g to assess the toxicity of the EH-EtOH. The experiment followed the guidelines provided by the OECD (Guideline No. 420). The mice were divided into four groups, each consisting of five animals. They were given a period of five days to acclimate before the test experiments, and they were fasted overnight prior to dosing. The first group served as the control and received a single dose of saline solution via oral gavage. While the remaining 3 experimental groups each received a single dose of the test material at doses of 500 mg/kg, 1000 mg/kg, and 2000 mg/kg, respectively, administered by oral gavage. Various physiological parameters such as the condition of their ears, skin, mucous membranes, eyes, respiration, circulatory system, autonomic responses, and somatomotor activity were observed for any signs of alteration. Specifically, behavior patterns related to convulsions, tremors, diarrhea, salivation, lethargy, sleep, and coma were closely monitored. The results indicated that the extract did not cause any fatalities or exhibit toxic indications at a dose of 2000 mg/kg body weight, suggesting its safety and non-toxic nature.

#### 2.4.3. Experimental animals for induced DM

About 48 male Albino Wister rats (Average weight 140–150 g) were purchased from the Animal Facility of the National Research Centre, Egypt. Animals were kept in standard cages, under pathogen-free conditions, and maintained under controlled room temperature and under normal dark–light cycles. The rats were allowed to adapt to these conditions for 2 weeks before beginning the experimental protocol. All studies were conducted in accordance with the Ethical Committee of the National Research Centre’s and Faculty of Veterinary Medicine, Cairo University [Approval No: Vet CU 09092023784] authorized Ethical Guidelines for the Care and Use of Experimental Animals.

The animals fed during the experiment on a normal Standard diet (AIN 1993) that consisted of the additional corn-soybean meal (44%)—corn gluten (62%)—barley—mixed vitamins, mineral salts—dicalcium phosphate—table salt—lysine monohydrochloride (98.5%)—methionine. With the specifications of Crude protein not less than 22%, total energy (kcal/kg) not less than 3948, crude fiber not less than 3.91, methionine not less than 0.66%, crude fat is not more than 3.5%, calcium and not less than 0.92%, lysine not less than 1.44%, phosphorus not less than 0.43%. and obtained from Ibex for the production of feed and fertilizers.

*2*.*4*.*3*.*1*. *Induction of diabetes*. Diabetes mellitus (DM) was induced by intraperitoneal injection (i.p.) of STZ 52 mg/kg dissolved in 0.1 M sodium citrate buffer (pH 4.5). The control group received an equivalent amount of buffer. After 3 days following STZ administration, blood was collected from the tail vein and analyzed for blood glucose level. Animals showing fasting blood glucose higher than 250 mg/dl were considered diabetic rats and used for the study.

Animals were randomly allocated into 6 groups (n = 8) as follows: **Group 1**, Normoglycemic control (healthy normal control): rats received normal saline solution (0.9%) for eight weeks. **Group 2** (positive control or DM-induced group) diabetic-induced that rat received 0.9% saline solution for 8 weeks [25]. **Group 3** (MET): DM-induced rats received metformin (150 mg/kg body weight; p.o) daily for 8 weeks. **Groups 4, 5 and 6** included the DM-induced rat that daily received EH-EtOH (50, 100, 200 mg/kg body weight; p.o) respectively for 8 weeks. After the experiment was over, all of the rats were quickly and mercifully sacrificed by cervical dislocation while under anesthesia with an intraperitoneal injection of 40–50 mg/kg of sodium pentobarbital. The rats were then disposed of in accordance with the guidelines set forth by the National Research Center’s Safety and Health Committee (NRC).

*2*.*4*.*3*.*2*. *Blood Collection and Tissue Preparation*. After a 48-hour period following the last treatment, the rats were fasted for at least 10 h before being anesthetized for blood sample collection. The blood was obtained from the caudal vein and collected in clean centrifuge tubes. It was then left to coagulate and subsequently centrifuged at 3000 rpm for 10 min using a cooling centrifuge from Sigma Laborzentrifugen GmbH (model 2-16KL; Osterode am Harz, Germany). The resulting serum was separated and stored in Eppendorf tubes at a temperature of -80 °C for future analysis of hormones (T4 and E2) as well as glucose levels. The testes of the rats were carefully dissected after euthanization through cervical dislocation and thoroughly cleansed using phosphate buffer saline (PBS) buffer. For the histological analysis, a portion of the testicular tissues from a predetermined number of animals in each group was fixed in a 10% formalin buffer for 24 h. The remaining parts of the tissues were homogenized in ice-cold 10% (*w/v*) phosphate buffer. The homogenate was then centrifuged at 1800 xg for 10 min at 4 °C, and the resulting supernatant was transferred to an Eppendorf tube kept at a low temperature for further measurement of other biochemical parameters [26].

*2*.*4*.*3*.*3*. *Preparation of testicular tissue extract*. The testes and accessory glands were homogenized in 20% (w/v) phosphate buffer saline (PBS), using an ultrasonic homogenizer. At 4 °C for 10 min, the homogenate underwent centrifugation at a speed of 1800× g. The resulting supernatant was carefully transferred into Eppendorf tubes and stored at -20°C for future use in the analysis of biochemical parameters.

*2*.*4*.*3*.*4*. *Assessment of testicular tissue antioxidant markers*, *Catalase*, *GSH and UGTs*. The quantitative determination of catalase, reduced glutathione (GSH), & UDP-glucoronosyl transferase (UGT) were assessed in testicular tissue homogenate using ELISA kits following the manufacturer’s instructions (SUNLONG BIOTECH CO., LTD, Hangzhou, China).

*2*.*4*.*3*.*5*. *Determination of testicular tissue inflammatory markers*. The levels of COX-2, PGE-2, IL-1*β*, and TNF-*α* were quantitatively measured using the ELISA technique. Specific kits from Sunlong Biotech Co., Ltd, (Hangzhou, China), were used for the analysis, following the instructions provided by the manufacturer.

*2*.*4*.*3*.*6*. *Assessment of testicular tissue content of VEGF*. The levels of VEGF were quantitatively assessed using ELISA method. The analysis was conducted according to the guidelines provided by the manufacturer (Sunlong Biotech Co., Ltd, located in Hangzhou city, China).

*2*.*4*.*3*.*7*. *Determination of serum levels of E2*, *T4*, *and glucose*. The serum T4 and E2 levels were determined by using commercial ELISA kits following manufacturer’s instructions. (Bios, San Diego, California, USA). While the serum glucose levels were colorimetrically determined using a glucose kit (DiaSys Diagnostic Systems GmbH, Germany) according to [27].

*2*.*4*.*3*.*8*. *Histopathological and immunohistochemical evaluation*. The testes were dehydrated in various alcohol grades, cleared in xylol, and embedded using paraffin following the standard procedures for formalin-fixed specimens of the testes. After the paraffin blocks were made, serial sections were cut and stained with Hematoxylin and Eosin (H&E) [28]. Moreover, Paraffin-embedded rat testicular tissues were deparaffinized. A primary anti-caspase-3 antibody (Abcam, Cambridge, MA, USA, ab184787) was then added at a dilution of 1:100, and the samples were incubated overnight at 4 °C. The slides were then incubated with a rabbit-specific secondary antibody conjugated to horseradish peroxidase. The slides were then hematoxylin-counterstained afterwards [29].

### 2.5. Statistical analysis

The results were presented as mean ± SD. To assess the significance of the mean differences between the groups, data analysis was conducted using one-way analysis of variance (ANOVA), followed by Tukey’s multiple comparison test. A *p*-value < 0.05 was considered statistically significant. The statistical analysis was performed using GraphPad Prism software (version 9.00, “GraphPad Software, LLC., San Diego, USA”).

## 3. Results

### 3.1. *E*. *heterophylla* metabolites profiling *via* UPLC–ESI-MS/MS

**Fig 1:**
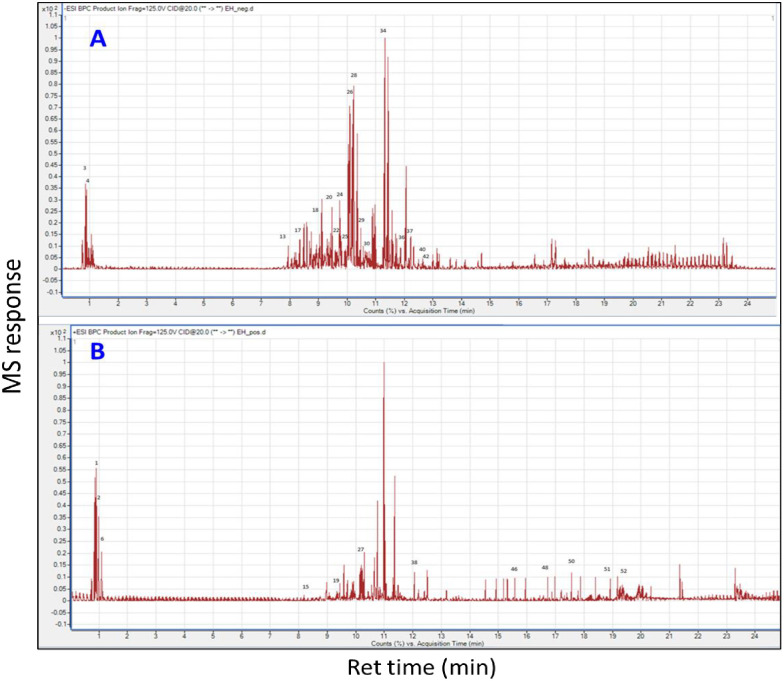
UPLC-MS chromatograms of ethanol extract of *Euphorbia heterophylla*. **A**: negative ion mode chromatogram. **B**: positive mode chromatogram.

**Fig 2:**
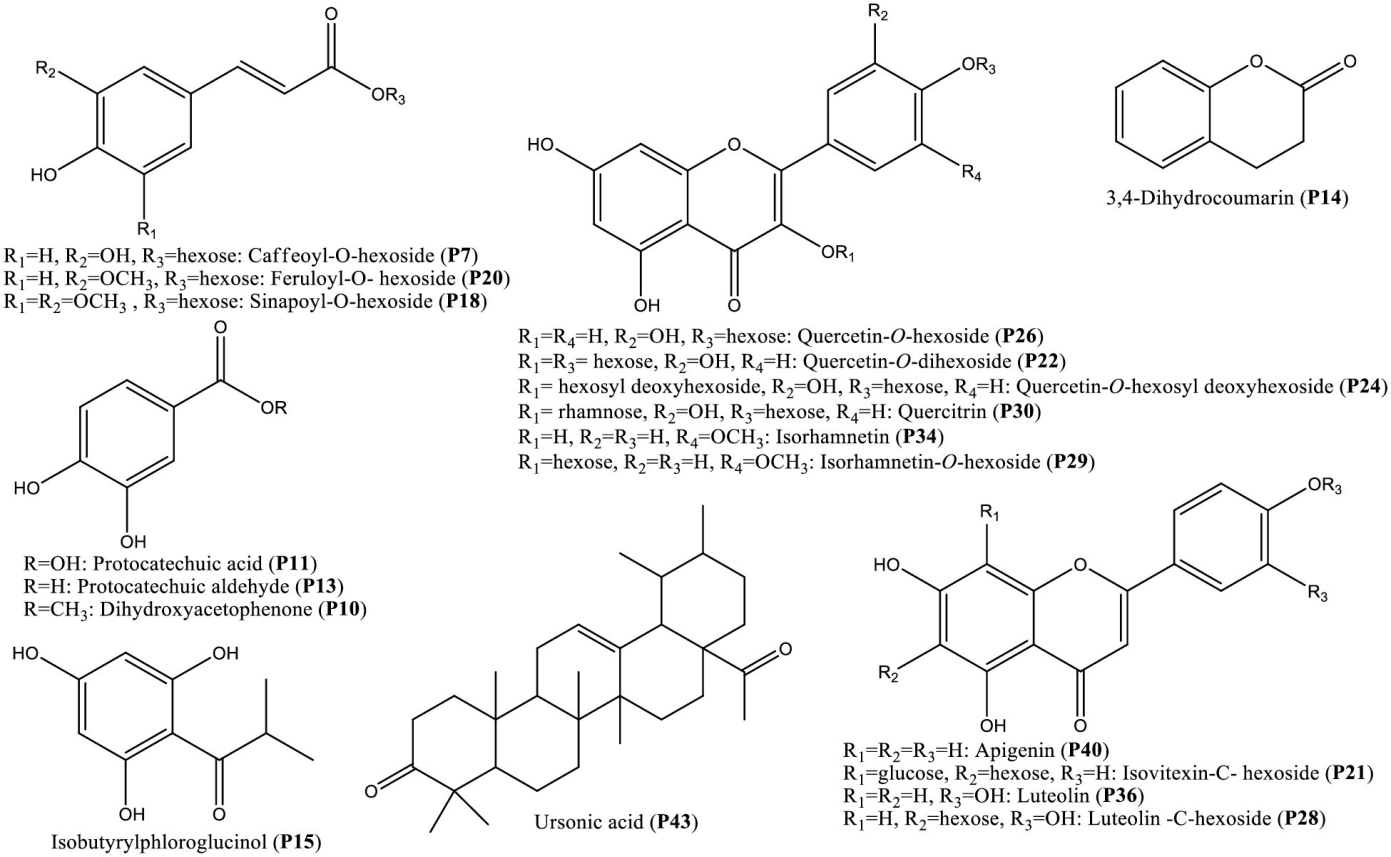
Chemical structures of the major identified compounds from *E*. *heterophylla* ethanolic extract.

*E*. *heterophylla* whole plant ethanolic extract was determined using high-resolution UHPLC-ESI-Orbitrap-MS operated in negative and positive electrospray ionization (ESI) modes to provide a comprehensive overview of *E*. *heterophylla* metabolome [30]. The metabolites were eluted in order of decreasing of the polarity as typical from reverse phase column. A total of 653 mass peaks were extracted in the positive ESI mode *vs*. 478 mass peaks in the negative ESI mode. Metabolite annotation was based on comparing retention time and MS data (accurate mass, molecular formula, isotopic distribution, and fragmentation pattern) of the detected compounds with those previously reported for *Euphorbia* alongside searching Sirius database version 5.6.2 https://bio.informatik.uni-jena.de/software/sirius/. The retention times, molecular formulae, observed molecular/ fragment ions, and metabolite identities are listed in Table 1. A total of 52 peaks were annotated belonging to sugars, sugar alcohols, sugar acids, organic acids, phenolic acids, flavonoids, coumarins, phloroglucinols, triterpenes, and fatty acids. Twenty-two compounds were detected in positive ion mode mostly belonging to coumarins, phloroglucinols, triterpenes, and fatty acids *vs*. thirty compounds identified in negative ion mode, chiefly flavonoids, and phenolic acids as listed in Table 1. The *E*. *heterophylla* UPLC-MS chromatograms alongside structures of major identified metabolites are presented in Figs 1 and 2, respectively. Metabolites were eluted in order of decreasing polarity that is, *i*.*e*., phenolic acids and/or flavonoid di-glucosides eluting first, followed by flavonoid mono-glucosides, flavonoid aglycones, eventually triterpenes, and fatty acids.

**Table 1:** Metabolites identified via UPLC-MS in *E*. *heterophylla* extract using negative and positive ionization modes.

Peak	Rt (min.)	Identification	Mol. ion *m/z*		Formula	Error (ppm)	MS/MS	Class
			[M-H]^−^	[M+H]^+^				
1	0.863	Sorbitol		183.0865	C_6_H_14_O_6_^+^	-0.94	85, 69, 61 57	Sugar alcohol
2	0.885	Fucose		165.0759	C_6_H_12_O_5_^+^	-0.46	85, 69, 57	Sugar
3	0.909	Ribonic acid	165.0412		C_5_H_9_O_6_^-^	-4.41	87, 75, 59	Sugar acid
4	0.913	Gluconic acid	195.0518		C_6_H_11_O_7_^-^	-3.59	-	Sugar acid
5	1.03	Malonic acid	103.0037		C_3_H_3_O_4_^-^	-0.57	86, 75, 59	Organic acid
6	1.106	Deoxythreitol		107.0702	C_4_H_10_O_3_^+^	4.33	79, 77, 94, 53	Sugar alcohol
7	1.156	Caffeoyl-*O*-hexoside	341.0883		C_15_H_17_O_9_^-^	-1.3	179, 161, 135	Phenolic acid
8	1.293	Vanillyl alcohol hexoside	315.1089		C_14_H_19_O_8_^-^	-1.22	136	Phenolic
9	2.392	*p*-Coumaric acid		165.0544	C_9_H_8_O_3_^+^		147, 121	Phenolic acid
10	2.613	Dihydroxyacetophenone	151.0403		C_8_H_7_O_3_^-^	-1.79	136, 123, 109, 108, 117	Phenolic
11	6.137	Protocatechuic acid	153.0197		C_7_H_5_O_4_^-^	-3.51	109	Phenolic acid
12	7.77	Unknown	373.1146		C_16_H_21_O_10_^-^	-1.29	211, 193,167,149, 123, 89	Unknown
13	7.85	Protocatechuic aldehyde	137.0246		C_7_H_5_O_3_^-^	-1.85	119, 108, 93	Phenolic acid
14	7.805	Dihydrocoumarin		149.0596	C_9_H_8_O_2_^+^	0.6	121, 105, 91, 79	Coumarin
15	8.285	Isobutyrylphloroglucinol		197.0806	C_10_H_12_O_4_^+^	0.99	180, 179, 169, 127	Phloroglucinol
16	8.288	Guaiacylglycerol		215.0912	C_10_H_14_O_5_^+^	-0.18	197, 179	Phenolic
17	8.464	Unknown	375.1304		C_16_H_23_O_10_^-^	-1.94	213, 169, 151	Unknown
18	9.048	Sinapoyl-*O*-hexoside	385.1147		C_17_H_21_O_10_^-^	-1.75	325, 223, 193, 179, 163, 162, 161, 135	Phenolic acid
19	9.277	Dihydroxy methoxy methylacetophenone-*O*-hexoside		359.1338	C_16_H_22_O_9_^+^	-0.19	342, 197, 180, 163, 133	Phloroglucinol
20	9.378	Feruloyl-*O*-hexoside	355.1043		C_16_H_19_O_9_^-^	-2.05	193, 175, 149	Phenolic acid
21	9.448	Isovitexin-*C*-hexoside	593.1516		C_27_H_29_O_15_^-^	-0.9	503, 473, 431, 413, 383, 353, 341, 268	Flavone-*C*-glycoside
22	9.637	Quercetin-*O*-dihexoside	625.1415		C_27_H_29_O_17_^-^	-0.6	463, 301, 179	Flavonol-*O*-glycoside
23	9.659	Unknown		675.2457	C_37_H_38_O_12_^+^	-3.08	658, 657, 644	Unknown
24	9.773	Quercetin-*O*-hexosyl deoxyhexoside	609.1468		C_27_H_29_O_16_^-^	-1.92	447, 301	Flavonol-*O*-glycoside
25	9.906	Acetyl vanillic acid	209.0459		C_10_H_9_O_5_^-^	-1.82	167, 134, 133, 123	Phenolic acid
26	10.133	Quercetin-*O*-hexoside	463.0889		C_21_H_19_O_12_^-^	-1.59	301, 191, 179, 151	Flavonol-*O*-glycoside
27	10.217	Dimethoxy benzoic acid		183.0651	C_9_H_10_O_4_^+^	0.66	168, 109, 107	Phenolic acid
28	10.255	Luteolin-*C*-hexoside	447.0939		C_21_H_19_O_11_^-^	-1.22	429, 357, 327, 285, 151	Flavone-*C*-glycoside
29	10.502	Isorhamnetin-*O*-hexoside	477.1043		C_22_H_21_O_12_^-^	-1.15	315	Flavonol-*O*-glycoside
30	10.699	Quercitrin	447.0942		C_21_H_19_O_11_^-^	-1.22	314, 301	Flavonol-*O*- glycoside
31	10.714	Unknown	637.2138		C_30_H_37_O_15_^-^	0.01	591, 461, 301, 175	Unknown
32	10.787	Unknown		149.0596	C_9_H_8_O_2_^+^	0.79	121, 103, 93, 91, 77	Unknown
33	10.877	Halfordin	275.0565		C_14_H_11_O_6_^-^	-1.45	260, 232, 217,189, 174	Coumarin
34	11.302	Isorhamnetin	315.0517		C_16_H_11_O_7_^-^	-1.83	300, 271, 255, 135	Flavonol
35	11.315	Unknown	651.23		C_31_H_39_O_15_^-^	-0.99	475, 315, 193, 175	Unknown
36	11.871	Luteolin	285.041		C_15_H_9_O_6_^-^	-1.72	243, 241, 199, 175, 133, 151	Flavone
37	12.044	Diosmetin	299.0569		C_16_H_11_O_6_^-^	-2.4	284, 256, 136	Flavone
38	12.078	Bicoumol		323.0524	C_18_H_10_O_6_^+^	8.21	163, 135, 119	Coumarin
39	12.101	Unknown	723.3236		C_36_H_51_O_15_^-^	-0.98	691, 368	Unknown
40	12.606	Apigenin	269.0463		C_15_H_9_O_5_^-^	-2.22	151, 117	Flavone
41	12.61	Unknown		351.2138	C_20_H_30_O_5_^+^	7.23	275, 244, 243, 201	Unknown
42	12.653	Trihydroxy-octadecadienoic acid	327.2182		C_18_H_31_O_5_^-^	-0.55	315, 309, 291, 229, 211, 171	Fatty acid
43	12.738	Ursonic acid		455.3515	C_30_H_46_O_3_^+^	1.45	411, 410, 409, 218, 203, 201, 189, 191, 131	Triterpene
44	13.449	Unknown		465.2452	C_25_H_36_O_8_^+^	6.6	245, 243, 131	Unknown
45	13.953	Dihydroxy-trimethoxy flavone	343.0827		C_18_H_15_O_7_^-^	-1.27	328, 313, 316, 315, 298	Flavone
46	15.624	Hexadecadienoic acid		253.216	C_16_H_28_O_2_^+^	1.11	209, 197, 141, 140, 127	Fatty acid
47	16.479	Monopalmitin		331.2839	C_19_H_38_O_4_^+^	0.87	314, 313, 257, 95, 81, 71, 57	Fatty acid ester
48	16.698	Octadecenedioic acid		313.2343	C_18_H_32_O_4_^+^	9.54	254, 164	Fatty acid
49	16.825	Unknown triterpen		703.3871	C_35_H_58_O1_4_^+^	4.48	430, 347	Triterpene
50	17.588	Unknown		143.1065	C_8_H_14_O_2_^+^	1.46	125, 99, 98, 84, 71, 70, 62	Unknown
51	18.809	Epoxy-octadecenoic acid		297.2397	C_18_H_32_O_3_^+^	9.08	184, 109	Fatty acid
52	19.374	Octadecadienoyl glycerol		355.2817	C_21_H_38_O_4_^+^	7.57	208	Fatty acid ester

#### 3.1.1. Phenolic acids and their derivatives

Phenolic acids are precursors for most phenolics typically conjugated with organic acids and or sugars. They appeared in the first part of the chromatogram in the negative ion mode. Their MS fragmentation was characterized by the loss of CO_2_ (-44 amu) and/or H_2_O (-18 amu) (Table 1) [31]. Three peaks (P 7, 18 & 20) showed the characteristic loss of hexose (-162 amu) with molecular ion peaks [M-H]^-^ at *m/z* (341.0883, 385.1147, and 355.1043, respectively) assigned as caffeoyl-*O*-hexoside (S1 Fig), sinapoyl-*O*-hexoside, and feruloyl-*O*- hexoside. Also, *p*-coumaric acid (P9), protocatechuic acid (P11, S2 Fig), protocatechuic aldehyde (P13), acetyl vanillic acid (P25), and dimethoxy benzoic acid (P27) were identified based on their mass spectral data (Table 1), in accordance with those of previous studies. Mainly, protocatechuic aldehyde, sinapoyl hexoside, feruloyl hexoside, and dimethoxy benzoic acid (P 13,18, 20, and 27) were the major phenolic acid derivatives annotated as represented in Fig 1. Of the detected phenolic acids and their derivatives, only *p-*coumaric acid has been previously reported in *E*. *heterophylla* [32], while feruloyl-*O*-hexoside, protocatechuic acid, *p*-coumaric acid, sinapoyl-*O*-hexoside, and caffeoyl-*O*-hexoside have been previously reported in different *Euphorbia* species [33, 34] to the best of our knowledge.

#### 3.1.2. Flavonoids

Flavonoids have been commonly reported from *Euphorbia* [35]. The current study provided an overview of the main flavonoids in *E*. *heterophylla* including 6 flavonols (P22, 24, 26, 29, 30, 34) and 5 flavones (P21, 28, 36, 37, 40). Where the major flavonoids were represented at (P 21, 22, 26, 28, 29, 30, 34, 36, 37, and 40) as displayed in Fig 1. In *Euphorbia*, the most abundant classes of flavonoids are flavonols and flavones with *O*-substitution patterns, *C*-methylation, and usually attached with glycosides at either C-3 or C-7 sites [36].

Using MS/MS analysis, the sugar type could be distinguished based on the neutral loss of 162 amu and 146 amu corresponding to hexose and deoxyhexose, respectively in the case of *O*-type glycosides [37]. While the fragmentation of *C*-glycosyl flavonoids is often characterized by the loss of water molecule [-18 amu] and cross-ring cleavage of the sugar (0,2 and 0,3), resulting in the loss of 120 and 90 amu, respectively in the case of *C*-hexosides [38]. A total of 7 mono- and di-flavonoid glycoside peaks were assigned (P21, 22, 24, 26, 28, 29, 30), *viz*. 5 flavonol and 2 flavone glycosides, based on their MS spectra.

*3*.*1*.*2*.*1*. *C-Flavonoid glycosides*. The detected *C*-flavonoid glycosides were mainly of the flavone type *viz*. apigenin and luteolin glycosides (P21 and P28) identified based on aglycone apigenin and luteolin fragment ions [M-H]^-^ at *m/z* 269 and 285, respectively. For example, P21 [*m/z* 593.1516 (C_27_H_30_O_15_)] showed characteristic fragment ions at *m/z* 503, 473, 413, and 383 after the subsequent loss of (90 and 120 amu) for the 2 sugar moieties and was readily assigned as isovitexin-*C*-hexoside (S3 Fig). Its monoglycoside, isovitexin, was previously detected in *Euphorbia hirta* [39]. Moreover, P28 at *m/z* 447.0939, C_21_H_20_O_11_ yielded fragment ions at *m/z* 429 [M-H-H_2_O]^-^, 357 [M-H-90]—, 327 [M-H-120]—and 151 due to ^1,3^A^-^ -retro-Diels-Alder fragmentation (RDA) [40] was identified as luteolin-*C*-hexoside.

*3*.*1*.*2*.*2*. *O-Flavonoid glycosides*. Unlike *C-*glycosides, whose fragmentation is primarily in the attached sugar part, *O*-type glycosides readily cleave sugar moiety upon fragmentation, *i*.*e*., losing 162 or 146 amu for hexose or deoxyhexose, respectively. Such a pattern was observed in 4 flavonol peaks containing quercetin aglycone (*m/z* 301, C_15_H_10_O_7_) as in (P22, P24, P26 & P30) annotated as quercetin-*O*-dihexoside (S4 Fig), quercetin-*O*-hexosyl deoxyhexoside, quercetin-*O*-hexoside (S5 Fig), and quercitrin respectively. The successive loss of 2 hexosyl moieties (-162) was observed in (P22) affording fragment ions at *m/z* 463 and 301, asides from *m/z* 179 and 121 corresponding to quercetin RDA ^1,2^A^-^ and ^1,2^B^-^ [40], respectively and characterized as quercetin-*O*-di-hexoside. Additionally, (P26) displayed a hexosyl loss yielding quercetin aglycone and its RDA fragmentation, in addition to daughter ion at *m/z* 151 standing for RDA ^1,2^A-CO, assigned as quercetin-*O*-hexoside. While the subsequent hexosyl and deoxyhexosyl loss (-162 + 146 amu) from quercetin aglycone was observed in (P24) detected as quercetin-*O*-hexosyl deoxyhexoside. Also, P30 showed a molecular ion peak at *m/z* 447.0942, C_21_H_20_O_11_ and yielded quercetin aglycone post the loss of deoxyhexosyl moiety and assigned as quercitrin (quercetin-*O*-rhamnoside). Likewise, isorhamnetin-*O*-hexoside (P29), a methylated analogue of quercetin was detected at *m/z* 477.1043 [M-H]^-^ from its aglycone fragment ion at *m/z* 315 (C_16_H_11_O_7_) post the loss of hexose moiety.

Among these flavonoid glycosides, only quercetin-*O*-hexosyl deoxyhexoside, quercetin-*O*-hexoside, and quercitrin have been previously reported in *E*. heterophylla [41, 42], besides this is the first characterization of isovitexin-*C*-hexoside, and isorhamnetin-*O*-hexoside in *Euphorbia* species. The rest of the identified flavonoids have been detected in many *Euphorbia* species *e*.*g*., *E*. *lanata*, *E*. *lunulata*, *E*. *fischeriana*, *E*. *esula*, *E*. *humifusa*, *E*. *hirta*, and *E*. *condylocarpa* [36, 43].

*3*.*1*.*2*.*3*. *Flavonoid aglycones*. Isorhamnetin (P34) [44] belonging to flavonol, and 3 flavone aglycones, *viz*. luteolin (P36) [40], diosmetin (P37), and apigenin (P40) [45] were characterized from their MS spectral data and fragmentation pattern Table (1). This is the first report of diosmetin in *Euphorbia* species using UPLC-MS. While isorhamnetin, luteolin and apigenin have been detected in *Euphorbia* species *e*.*g*., *E*. *lanata*, *E*. *lunulata*, *E*. *humifusa*, *E*. *hirta*, and *E*. *condylocarpa* [36, 43]

#### 3.1.3. Miscellaneous phenolics

*3*.*1*.*3*.*1*. *Coumarins*. Peak (P14) with [M + H]^+^ at *m/z* 149.0596, C_9_H_8_O_2_ was annotated as dihydrocoumarin. Upon fragmentation, it yielded an ion at *m/z* 105, corresponding to CO_2_ loss from the pyrone ring system, followed by another one at *m/z* 79 corresponding to the loss of C_2_H_2_ [46]. Another coumarin was detected in a major peak (P38) with [M + H]^+^ at *m/z* 323.0524, C_18_H_10_O_6_ was assigned as bicoumol (S6 Fig) with characteristic fragment ions at *m/z* 163 (C_9_H_7_O_3_) corresponding to hydroxy coumarin, followed by the loss of CO (-28 amu) yielding ion at *m/z* 135, and another one at *m/z* 119 due to the loss of CO_2_ (-44 amu) [47]. Halfordin (P33) has been identified for the first time in *Euphorbia* having molecular ion peak [M-H]^-^ at *m/z* 275.0565, C_14_H_12_O_6_. On fragmentation, it yielded daughter ions at *m/z* 260, 232, 217, 189, and 174 corresponding to [M-H-CH_3_]^-^, [M-H-CH_3_-CO]^-^, [M-H-CH_3_-CO-CH_3_]^-^, [M-H-CH_3_-CO-C_2_H_3_O]^-^, and [M-H-CH_3_-CO-C_2_H_3_O-CH_3_]^-^, respectively. Several coumarins have been reported from *Euphorbia* as bicoumol from the stem wood of *E*. *quinquecostata* [35], albeit this is the first characterization of *E*. *heterophylla*.

*3*.*1*.*3*.*2*. *Phloroglucinols*. Several phloroglucinol derivatives, including acetophenones and their glycosides, have been previously reported in *E*. *portulacoides*, *E*. *quinquecostata*, *E*. *quinquecostata*, *E*. *fischeriana*, and *E*. *ebracteolate* [35] and to account for several of the health benefits in that genus. Isobutyrylphloroglucinol (P15, S7 Fig) was assigned from its [M+H]^+^ at *m/z* 197.0806, C_10_H_12_O_4_. Upon fragmentation, it yielded characteristic ions at *m/z* 180, 179, and 169 corresponding to the loss of OH (-17 amu), H_2_O (-18 amu), CO (-28 amu), respectively. While the fragment ion appearing at *m/z* 127 was attributed to the loss of 70 amu corresponding to isobutyryl group (C_4_H_7_O) [48]. Dihydroxy methoxy methylacetophenone-*O*-hexoside (P19) with [M+H]^+^ at *m/z* 359.1338, C_16_H_22_O_9_produced fragment ions corresponding to the successive losses of hexosyl, hydroxyl, and methoxy moieties *i*.*e*., (- 162, 17, and 30 amu respectively) yielding daughter ions at *m/z* 342, 197, 180, 163, and 133 [49]. Both isobutyrylphloroglucinol and dihydroxy methoxy methylacetophenone-*O*-hexoside were major compounds in *E*. *heterophylla* extract as displayed in Fig 1. This is the first detection of both phloroglucinol derivatives in *E*. *heterophylla*, while dihydroxy methoxy methylacetophenone-*O*-hexoside has been previously reported in *E*. *ebracteolata* [50].

Other simple phenolics annotated included vanillyl alcohol-*O*-hexoside (P8), guaiacylglycerol (P16), and dihydroxyacetophenone (P10). This is the first report of vanillyl alcohol-*O*-hexoside in *Euphorbia*, albeit it has been previously reported in family Euphorbiaceae in *Antidesma ghaesembilla*, whereas guaiacylglycerol was previously detected in *E*. *mauritanica* [17].

#### 3.1.4. Fatty acids

Several fatty acids eluted late in the chromatogram (Rt 15.624–19.374 min) were identified based on their MS spectra. Three unsaturated fatty acids were annotated *viz*. hexadecadienoic acid (P46), octadecenedioic acid (P48), and octenoic acid (P50) exhibiting parent ions [M+H]^+^ at *m/z* (253.216, C_16_H_28_O_2_), (313.2343, C_18_H_32_O_4_), and (143.1065, C_8_H_14_O_2_), respectively. Their fragmentation showed typical losses of fatty acids manifested by the loss of (-H_2_O), (-CH_2_)_n_, (-COOH), (-CO_2_) (-CH_2_)_n_-CO_2_) [51]. For example, octenoic acid displayed fragment ions at *m/z* 125 [M+H-H_2_O]^+^, 99 [M+H -CO_2_]^+^, 98 [M+H -COOH]^+^, 71 [M+H -CO_2_-(CH_2_)_2_]^+^, 70 [M+H–(CH_2_)_5_]^+^.

Whereas an epoxy acid, epoxy-octadecenoic acid was annotated based on its molecular ion at *m/z* 297.2397, C_18_H_32_O_3,_ previously identified in *E*. *lagascae* seed [52], while other epoxy acids were detected in *E*. *heterophylla* [53].

In addition, a trihydroxylated fatty acid (P42) showed successive loss of three successive water molecules (-18 amu) from its [M-H]^-^ (*m/z* 327.2182, C_18_H_32_O_5_) assigned as trihydroxy-octadecadienoic acid. Hydroxy fatty were previously detected in *Euphorbia*, accounting for cytotoxic, antifungal, and antitubercular effects in that genus [54, 55]. Additionally, two fatty acid esters, monopalmitin (P47) and octadecadienoyl glycerol (P52) were annotated based on molecular ion peaks at *m/z* (331.2839, C_19_H_38_O_4_) and (355.2817, C_21_H_38_O_4_), respectively.

#### 3.1.5. Terpenes

Ursonic acid was annotated in (P43) [M+H]^+^ at *m/z* 455.3515, C_30_H_46_O_3_ showing fragment ions at *m/z* 411 [M+H -COOH]^+^, 410 [M+H -HCOOH]^+^, 409 [M+H-CH_2_O_2_]^+^, 218 due to RDA fragments, 203 corresponding to RDA and the loss of CH_3_), 201, 189, and 131 after RDA [56, 57]. Ursonic acid is similar in structure to ursolic acid, previously reported in many plants e.g., *Ziziphus jujuba* fruits, *Lantana tiliaefolia* leaves, and *Catharanthus Roseus* plant, and this is its first characterization of *Euphorbia* and whether it contributes to the plant biological effects i.e., anticancer, and antiprotozoal [58] should be determined.

### 3.2. Biological assays

#### 3.2.1. LD_50_ assay

Swiss albino mice were administered EH-EtOH orally at doses up to 2,000 mg/kg, although neither of these materials caused any visible toxicity or mortality within 24 hours. The median lethal dose (LD_50_) of EH-EtOH in mice may therefore be greater than 2,000 mg/kg. It was documented that the tested extract is considered safe and nontoxic within LD_50_ values greater than 50 mg/kg body weight. This outcome was entirely consistent with the data of Nalule, 2017 [59], who reported that the bio- and histochemical assays in mice showed that EH-EtOH is safe up to 4,000 mg/kg body weight.

#### 3.2.2. Effect of *E*. *heterophylla* on glucose serum level and body and testicular weight

**Fig 3:**
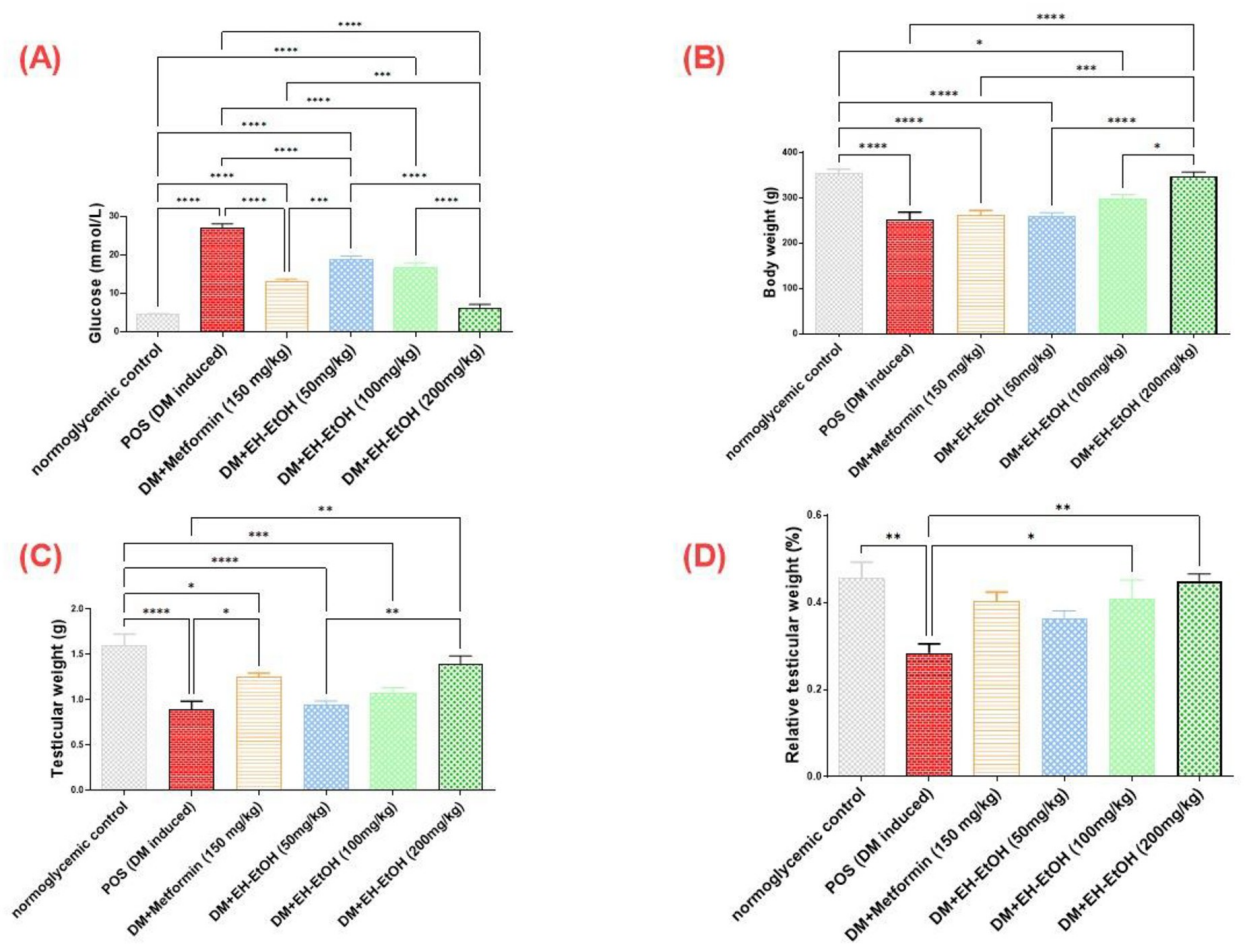
Effect of metformin (150 mg/kg.bw) and EH-EtOH (50, 100, 200 mg/kg.bw) on metabolic status and reproductive health. (**A**) Effect on the serum glucose levels (mmol/L), (**B**) Effect on the body weight (g), (**C**) Effect on the testicular weight (g). (**D**) Effect on the relative testicular weight (%). Data are expressed as (mean ± SD) where (n = 8). Statistical analysis was carried out by one-way analysis of variance (ANOVA) and followed by Tukey’s multiple comparison test. *p ≤ 0.05, **p ≤ 0.01, ***p ≤ 0.001, ****p ≤ 0.0001, POS is a positive group (DM-induced group), DM (Diabetes mellitus), (EH-EtOH) *E*. *heterophylla* ethanol extract, DM+metformin (DM-induced group treated with metformin).

The diabetic group exhibited a notable increase in serum glucose level compared with the control group (*p* < 0.0001). However, upon treatment of the diabetic rats with metformin (150 mg/kg) and EH-EtOH (50, 100 and 200 mg/kg), a significant reduction in serum glucose levels was observed in comparison with untreated diabetic rats (Fig 3A). Furthermore, the diabetic rats experienced a significant decrease in whole body and testicular weight (*p* < 0.0001) and the relative testicular ratio to body weight (*p* ≤ 0.01) when compared with the control group (Fig 3B–3D). A significant improvement in both body and testicular weights was detected in high dose of EH (200mg/kg) (*p* < 0.0001) surpassing even levels observed in the untreated diabetic rats (Fig 3B and 3C). while in metformin group the improvement *p ≤ 0.05 was recorded in testicular weight only. the significant recorded improvement in relative testicular ratio was shown in rats received EH in both (100, 200mg/BW) in dose gradient effect (Fig 3D).

#### 3.2.3. Effect of *E*. *heterophylla* on testicular tissue antioxidant markers, catalase, GSH and UGTs

**Fig 4:**
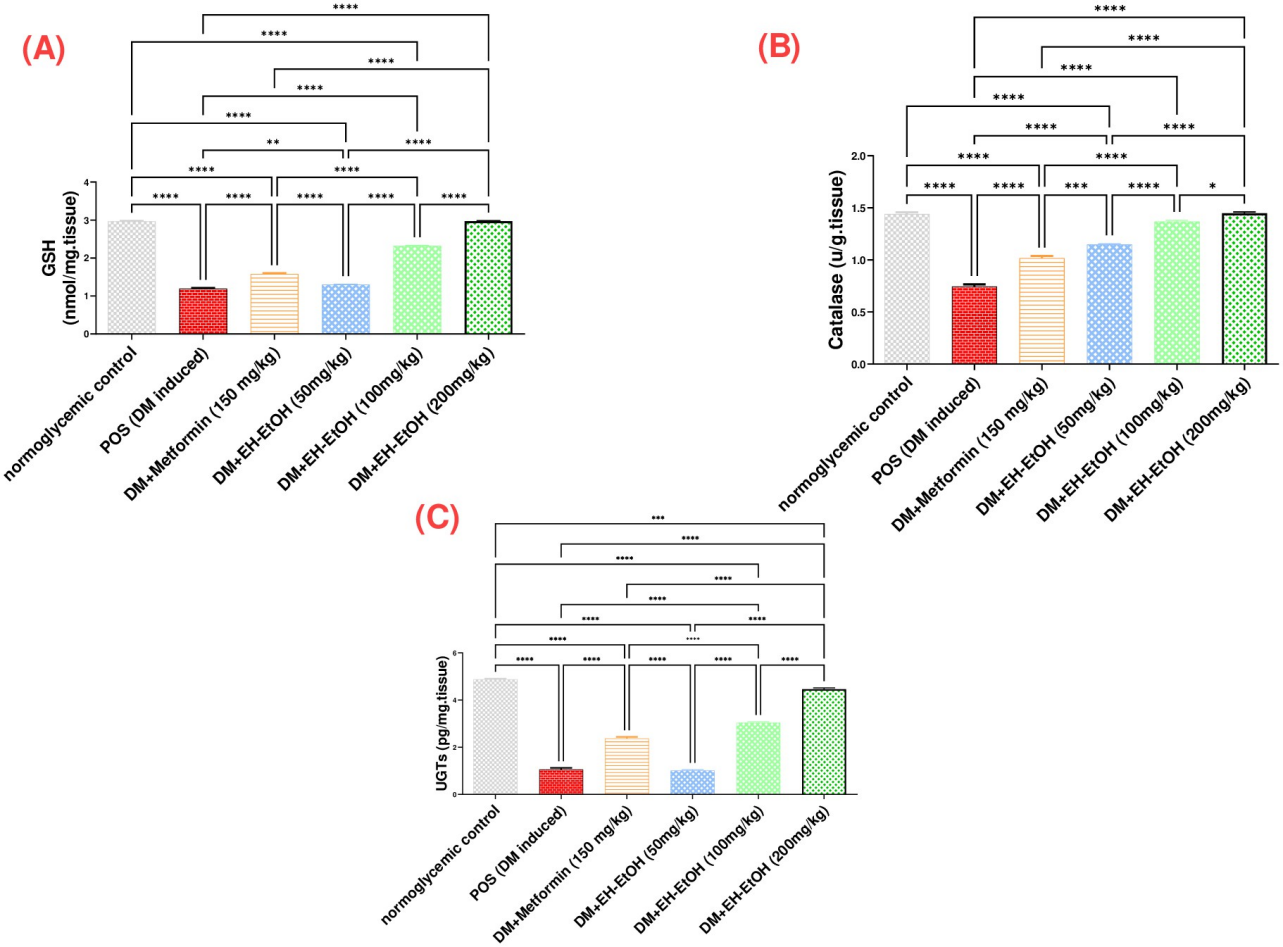
Effect of metformin (150mg/kg.bw) and EH-EtOH (50, 100, 200 mg/kg.bw) on testicular tissue antioxidant status. (**A**) Effect on Catalase. (**B**) Effect on GSH. (**C**) Effect on UGTs. Data are expressed as (mean ± SD) where (n = 8). Statistical analysis was carried out by one-way analysis of variance (ANOVA) and followed by Tukey’s multiple comparison test. *p ≤ 0.05, **p ≤ 0.01, ***p ≤ 0.001, ****p ≤ 0.0001, POS is a positive group (DM-induced group), DM (Diabetes mellitus), (EH-EtOH) *E*. *heterophylla* ethanol extract, DM+metformin (DM-induced group treated with metformin).

The levels of antioxidant biomarkers (GSH, Catalase, UGTs) were significantly reduced in the testicular tissue of the DM-induced group rats as compared with control rats (Fig 4A–4C) respectively. However, treatment with metformin (150 mg/kg) and EH-EtOH (50, 100 and 200 mg/kg) significantly increased the levels of catalase, GSH and UGTs compared with the DM-induced group rats (*p* < 0.0001). The best results were obtained with EH-EtOH (200 mg/kg) compared with the DM-induced group (*p* <0.0001) and metformin (*p* < 0.0001).

#### 3.2.4. Effect of *E*. *heterophylla* against inflammatory markers

**Fig 5:**
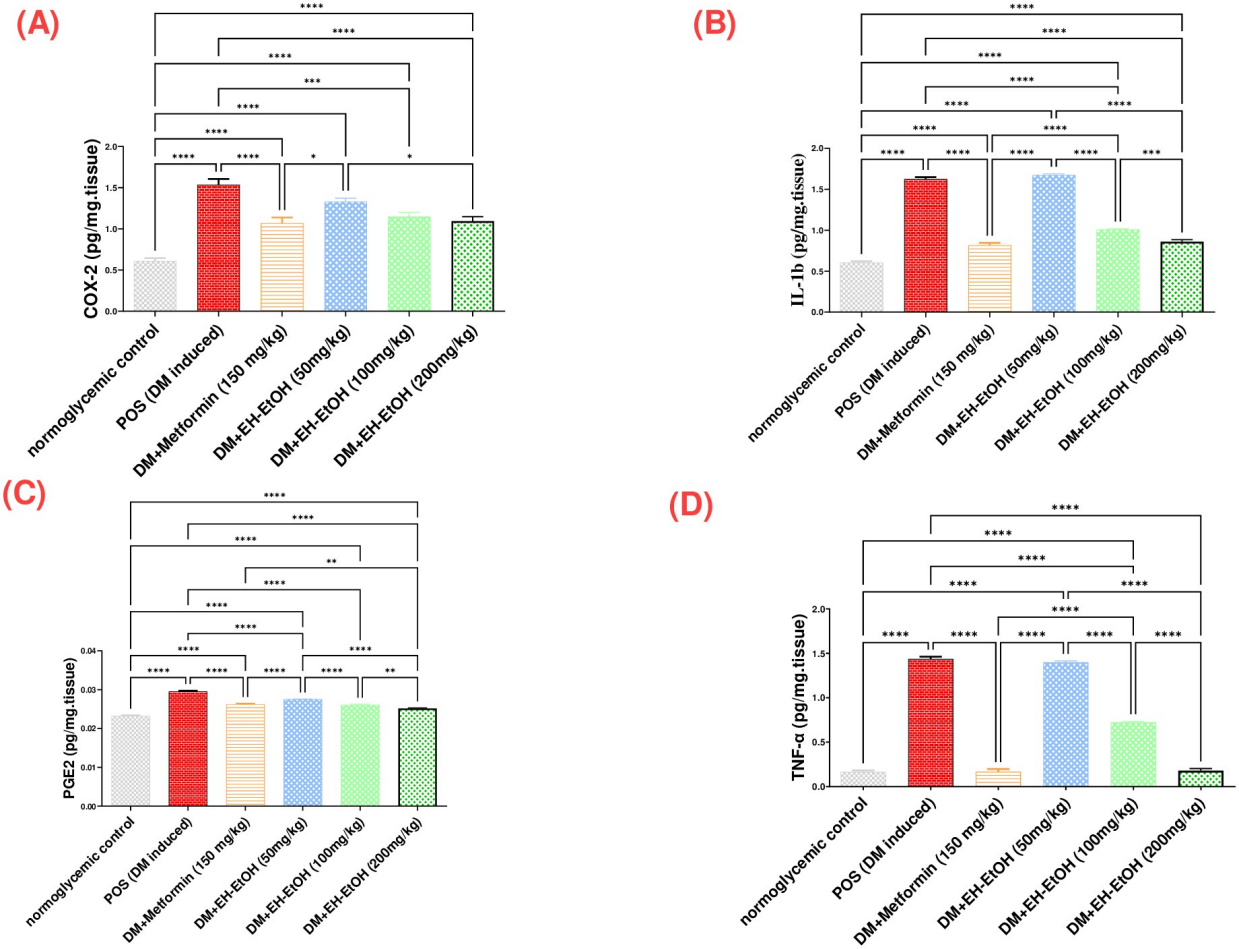
Effect of metformin (150 mg/kg.bw) and EH-EtOH (50, 100, 200 mg/kg.bw) on inflammatory status. (**A**) Effect on Cox-2 level. (**B**) Effect on IL-1*β* level. (**C**) Effect on PGE-2 level. (**D**) Effect on TNF-*α* level. Data are expressed as (mean ± SD) where (n = 8). Statistical analysis was carried out by one-way analysis of variance (ANOVA) and followed by Tukey’s multiple comparison test. *p ≤ 0.05, **p ≤ 0.01, ***p ≤ 0.001, ****p ≤ 0.0001, POS is a positive group (DM-induced group), DM (Diabetes mellitus), (EH-EtOH) *E*. *heterophylla* ethanol extract, DM+metformin (DM-induced group treated with metformin).

The levels of inflammatory biomarkers (COX-2, IL-1*β*, PGE-2 and TNF-*α*) were significantly increased in the testicular tissue of the DM-induced group rats as compared with control rats (*p* < 0.0001) as shown in (Fig 5A–5D), respectively. However, treatment with Metformin (150 mg/kg) and EH-EtOH (50, 100 and 200 mg/kg) significantly decreased the levels of COX-2, PGE-2, IL-1*β* and TNF-*α* compared with the DM-induced group rats (*p* < 0.0001). Among treatments, the best results were obtained with the dose EH-EtOH (200 mg/kg) compared with the DM-induced group (*p* < 0.0001).

#### 3.2.5. Effect of *E*. *heterophylla* on test*icular* tissue VEGF

**Fig 6:**
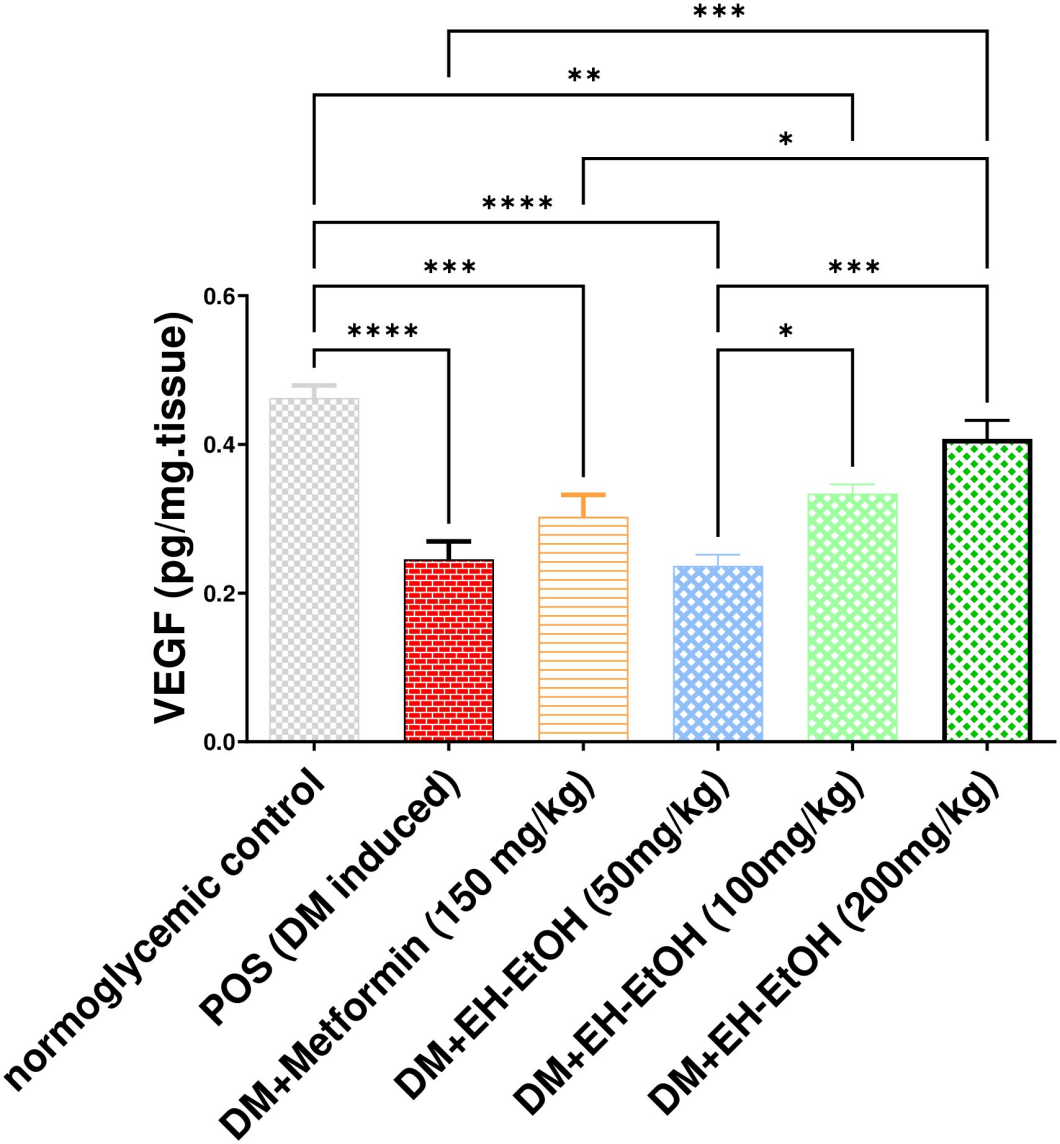
Effect of Metformin (150mg/kg.bw) and EH-EtOH (50, 100, 200 mg/kg.bw) on VEGF. Data are expressed as (mean ± SD) where (n = 8). Statistical analysis was carried out by one-way analysis of variance (ANOVA) and followed by Tukey’s multiple comparison test. *p ≤ 0.05, **p ≤ 0.01, ***p ≤ 0.001, ****p ≤ 0.0001, POS is a positive group (DM-induced group), DM (Diabetes mellitus), (EH-EtOH) *E*. *heterophylla* ethanol extract, DM+metformin (DM-induced group treated with metformin).

The VEGF level was significantly (*p* < 0.0001) reduced in the testicular tissue of the DM-induced group rats compared with control rats (Fig 6). However, treatment with Metformin (150 mg/kg) and EH-EtOH (50, 100 and 200 mg/kg) significantly increased the VEGF levels compared with the DM-induced group rats (*p* < 0.05). The best results were obtained in the case of EH-EtOH (200 mg/kg) compared with the DM-induced group (*p* < 0.001).

#### 3.2.6. Effect of *E*. *heterophylla* on serum levels of E2 and T4 hormones

**Fig 7:**
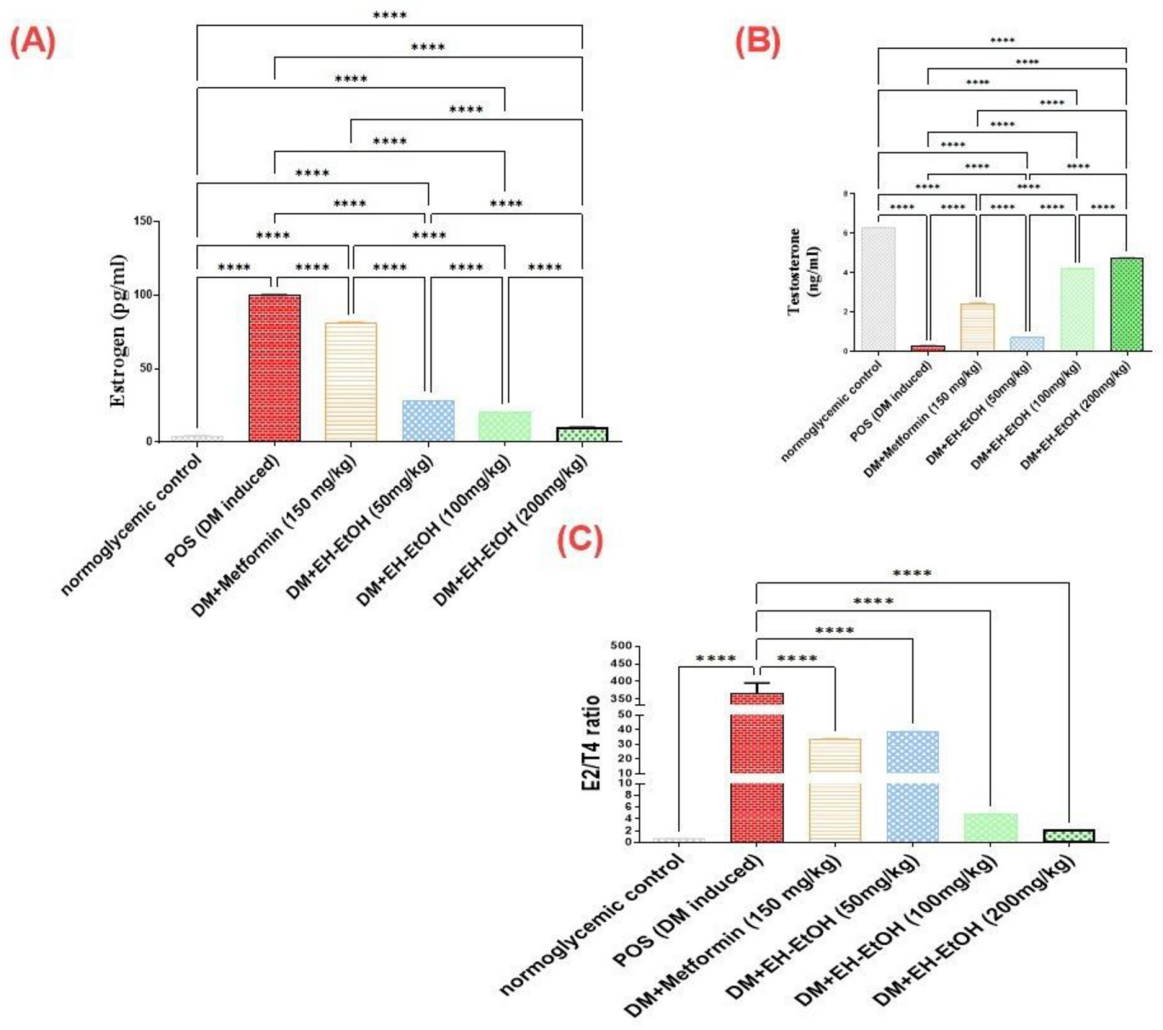
Effect of metformin (150mg/kg.bw) and EH-EtOH (50, 100, 200 mg/kg.bw) on hormonal status and reproductive health. (A) Effect on E2 (pg/ml) level. (B) Effect on T4 (ng/ml) level. (B) Effect on E2/T4 ratio. Data are expressed as (mean ± SD) where (n = 8). Statistical analysis was carried out by one-way analysis of variance (ANOVA) and followed by Tukey’s multiple comparison test. ****p ≤ 0.0001, POS is a positive group (DM-induced group), DM (Diabetes mellitus), EH-EtOH (*E*. *heterophylla* ethanol extract), DM+metformin (DM-induced group treated with metformin).

The T4 levels were significantly reduced in the testes tissue homogenate of the DM-induced group rats as compared with control rats (Fig 7), concurrent with increase in E2 levels (*p*<0.0001). A significant increase in T4 level was observed in the groups treated with metformin (*p*<0.0001) and EH-EtOH (50, 100 and 200 mg/kg) compared with the DM-induced group (*p*<0.0001). In contrast, a significant decrease of E2 levels was observed in the groups treated with metformin (*p*<0.0001) and EH-EtOH (50, 100 and 200 mg/kg) compared with the DM-induced group (*p*<0.0001). The highest decline in E2/T4 ratio (*p*<0.0001) was observed in the group treated with metformin and EH-EtOH while EH (200 mg/kg) was restored E2/T4 ratio to the basal levels that recorded in control group and in accordance with other bioassay results.

#### 3.2.7. Histopathological and immunohistochemical evaluation

**Fig 8:**
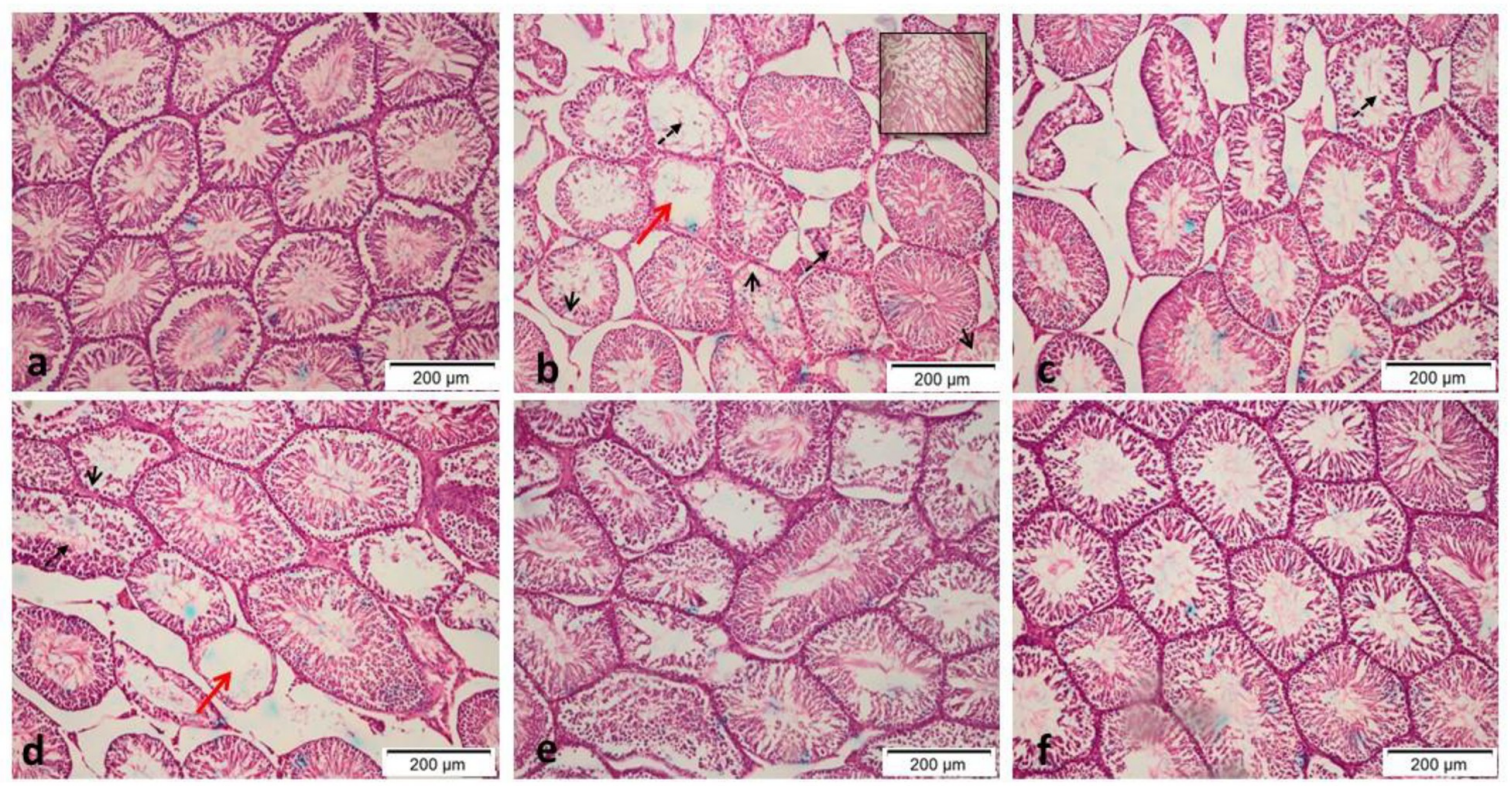
Representative histopathological images of H&E of testicular tissue of male rats. **a**: Normoglycemic control (N). **b**: Positive group (DM-induced) which showed large extensive degeneration with death of germ cells (dashed black arrow), as well as mild vacuolation of Sertoli cells (short black arrow) and Atrophy of seminiferous tubules (red arrow). **c**: Metformin (150 mg/kg body weight; p.o.) treated group which showed moderate degeneration and apoptosis of some germ cells in seminiferous tubules. All EH-EtOH groups revealed significant improvement when compared to POS group. **d**: EH-EtOH (Low dose, 50 mg/kg body weight; p.o.). **e**: EH-EtOH (Medium dose, 100 mg/kg body weight; p.o.). **f**: EH-EtOH (High dose, 200 mg/kg body weight; p.o.) revealed a superior effect in improving the histological condition of the testicular tissues. (Scale bar 200 μm).

**Fig 9:**
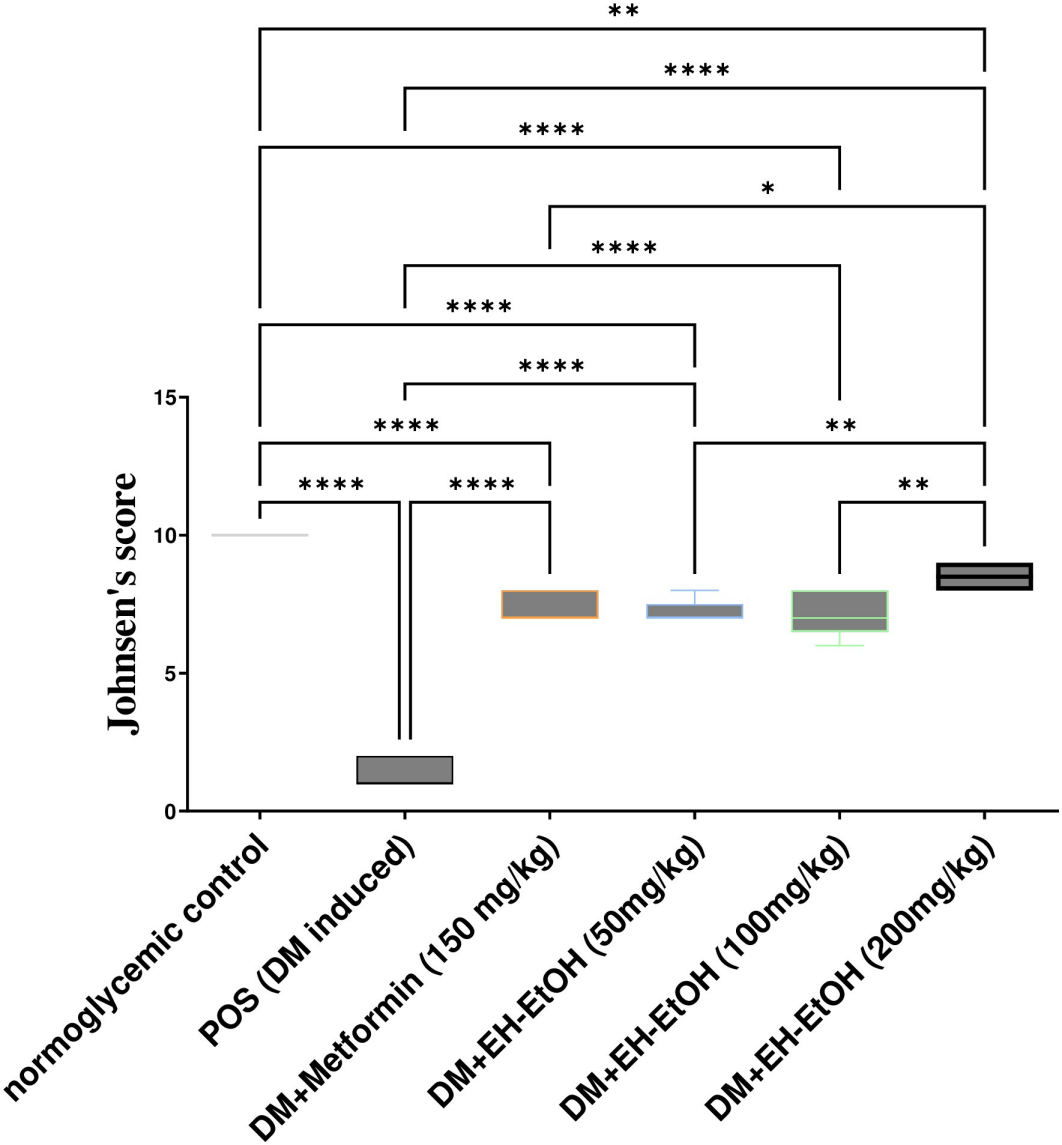
Johnsen’s score. The score ranges from 1 to 10, with each number representing a different stage of spermatogenesis and the presence or absence of specific cell types within the seminiferous tubules. Scoring data are presented as median (max–min) using the Kruskal–Wallis test followed by the Mann–Whitney U test. Data are expressed as nonparametrically (median ± IQR) where n = 8. Statistical analysis was performed using the one-way analysis of variance (ANOVA) followed by Tukey’s multiple comparison test. * *p* ≤ 0.05, ** *p* ≤ 0.01, *** *p* ≤ 0.001, **** *p* ≤ 0.0001. Where, POS is a positive group (DM-induced group), DM (Diabetes mellitus), EH-EtOH (*E*. *heterophylla* ethanol extract), DM+metformin (DM-induced group treated with metformin).

Control normoglycemic rats exhibited normal histological structure, with compactly packed seminiferous tubules and a synchronized population of mature germ cells (Fig 8a). In contrast, serious histological abnormalities with large extensive degeneration and death of germ cells, as well as mild vacuolation of Sertoli cells, were seen in the POS group (Fig 8b). Atrophy of seminiferous tubules with a decrease in Leydig cell number was a common lesion in this group. No significant difference between the effects of metformin (150 mg/kg body weight; p.o.) and EH-EtOH (50 mg/kg body weight; p.o.) was detected as shown in (Fig 8c and 8d) respectively, which revealed degeneration and apoptosis of some germ cells in seminiferous tubules with a lesser degree in comparison to the POS group. On the other hand, both groups treated with EH-EtOH at dosages (100 and 200 mg/kg body weight; p.o.) demonstrated a superior improvement in testicular tissue conditions in a dose-dependent manner (Fig 8e and 8f) respectively. These results revealed that EH-EtOH (200 mg/kg body weight; p.o.) (Fig 8f) exerted a superior effect in improving the histological condition of the testicular tissues. Also, Johnsen’s score (a histopathological grading system used to evaluate spermatogenesis in testicular biopsies) in the POS group were markedly (p < 0.0001) lower than that of the control negative and other treated groups (Fig 9). In EH-EtOH (200 mg/kg), they were effectively reversed. There was no discernible distinction between the DM+Metformin (150 mg/kg) and EH-EtOH (50 mg/kg) groups. These findings indicated that EH-EtOH (200 mg/kg body weight; p.o.) had a more advantageous impact on the histological condition of the testicular tissues.

#### 3.2.8. Caspase-3 protein expression in different treated groups

**Fig 10:**
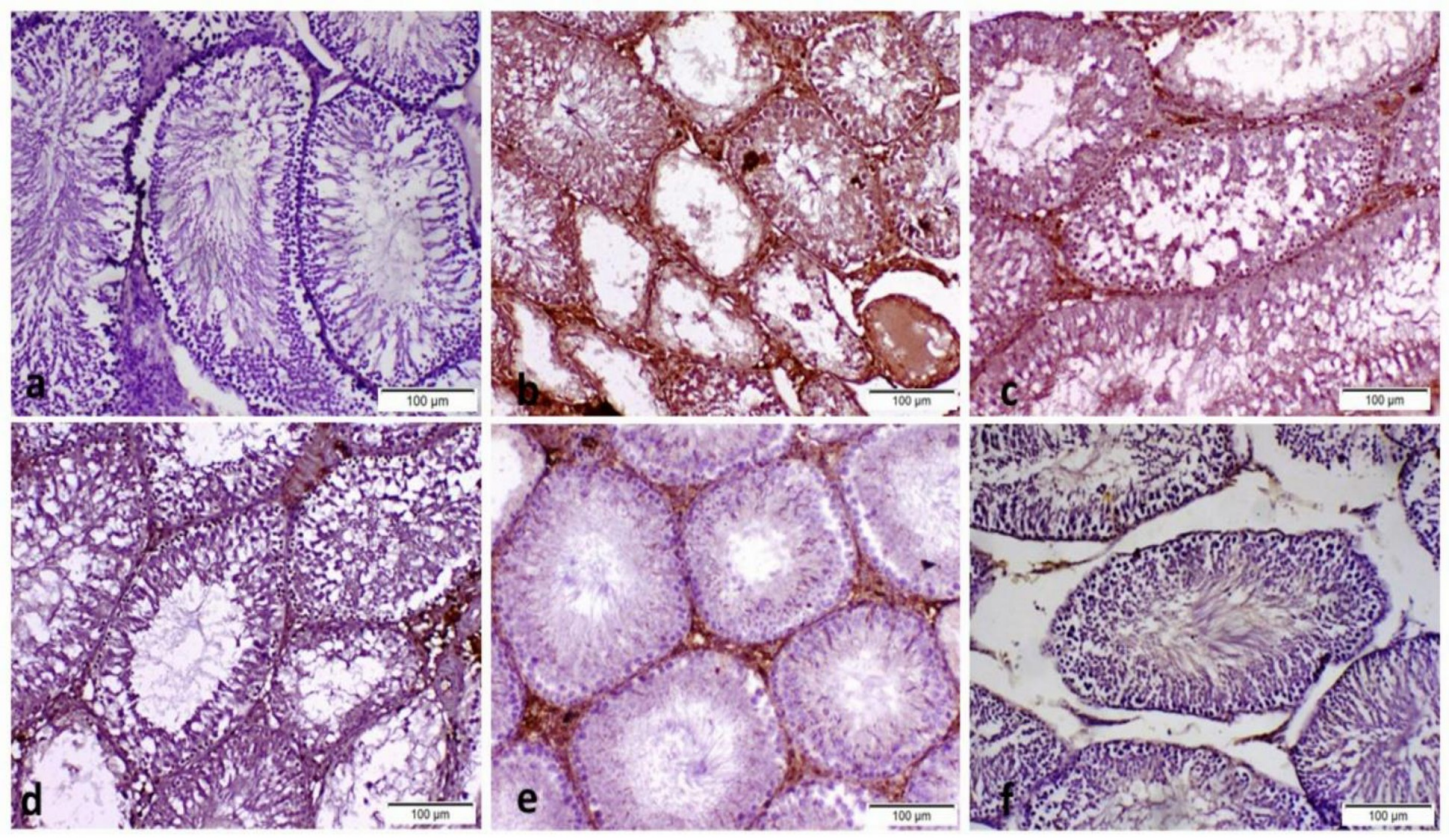
Photomicrographs of testicular tissue expression of cleaved caspase-3 immune-stained. **a**: Normoglycemic control (N) which showed negative expression. **b**: Positive group (DM-induced) which showed highly intense brown color expression. **c**: Metformin (150 mg/kg body weight; p.o.) treated group which showed moderate brown color expression. EH-EtOH groups showed decrease in the intensity of brown color expression. **d**: EH-EtOH (Low dose, 50 mg/kg body weight; p.o.). **e**: EH-EtOH (Medium dose, 100 mg/kg body weight; p.o.). **f**: EH-EtOH (High dose, 200 mg/kg body weight; p.o.) which showed the lowest brown expression when compared to all treated and control groups. (Scale bar 100 μm).

**Fig 11:**
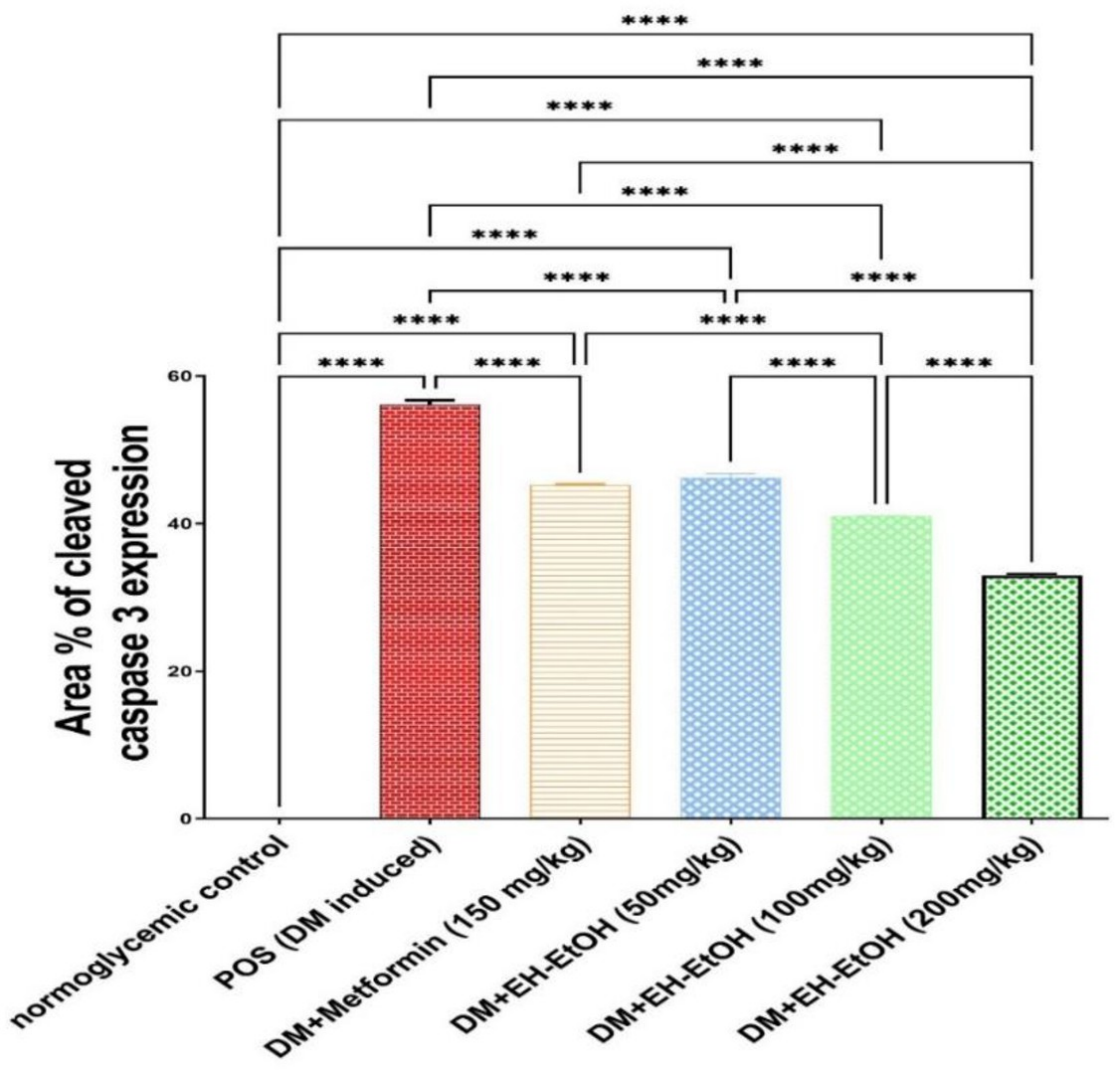
Cleaved caspase-3 expressed as area percent. Data are expressed as (mean ± SD) where (n = 8). Statistical analysis was carried out by one-way analysis of variance (ANOVA) and followed by Tukey’s multiple comparison test. ****p ≤ 0.0001, POS is a positive group (DM-induced group), DM (Diabetes mellitus), EH-EtOH (*E*. *heterophylla* ethanol extract), DM+metformin (DM-induced group treated with metformin).

No significant cleaved caspase-3 expression was demonstrated in the testes of normal control groups. In contrast, higher caspase-3 expression was found in POS rats, which showed a statistically superior elevation in gene expression (*p* ≤ 0.05). Caspase-3 expression was significantly reduced in rats treated with Metformin and three different doses of EH-EtOH (*p* ≤ 0.05) (Figs 10 and 11). In comparison to the control and other treated groups, the EH-EtOH (200 mg/kg body weight; p.o.) group showed the greatest drop in cleaved caspase-3 expression.

## 4. Discussion

This study revealed that DM induction using STZ caused testicular degeneration, characterized by changes in blood glucose levels, body weight, testicular inflammation, oxidative damage, and testicular cell apoptosis. In contrast, *E*. *heterophylla* ethanolic extract administration effectively mitigated these damages by restoring T4 levels, improving antioxidant capacity, reducing pro-inflammatory cytokine expression, and inhibiting testicular cell apoptosis. This highlights the importance of developing strategies to protect testicular health in diabetic males, especially considering its positive effects on fertility and reproductive functions. The STZ-induced model successfully replicated the features of DM, making it a suitable model for studying the effects of diabetes on testicular functions.

To establish a rat model of diabetes, the classical method of intraperitoneal (i.p.) injection of STZ, a genotoxic agent, was employed that has led to disrupting the balance between antioxidant and oxidant systems in pancreatic β cells. Consequently, it damages the insulin-producing islet *β*-cells, resulting in hyperglycemia and a significant decrease in insulin secretion within 48 h post-injection. The present study utilized STZ-induced diabetic rat model that successfully showed elevated serum glucose levels, decreased body and testicular weights compared with the control group [5]. In the current study, diabetic rats that received treatment with EH-EtOH demonstrated a significant increase in both body and testicular weights compared with the control group. Additionally, these rats exhibited a decrease in blood glucose levels. Furthermore, the histopathological patterns of the testicular tissue and the process of spermatogenesis that assessed by Johnsen’s score [60] were restored to normal levels in the case of EH-EtOH-treated group, contrasting with the diabetic rats that did not receive treatment. These outcomes are consistent with the previous reports [61]. The significant presence of protocatechuic aldehyde, sinapoyl-*O*-hexoside, feruloyl-*O*-hexoside, and other phenolic acids, along with flavonoids, played a crucial role in regulating blood glucose levels and managing body weight in diabetic cases. These effects are attributed to inhibitory action in the gut for glucose absorption or by its peripheral tissue uptake or the improvement of insulin resistance but the main action owed to the restoration of oxidative balance in type-2 diabetic rats [62, 63].

The excessive production of ROS induced by STZ is a critical pathway leading to oxidative stress. This oxidative stress results in decreased antioxidant activity and is considered the primary contributor to diabetic complications. These complications arise from the overproduction of free radicals during glucose auto-oxidation. [64]. Studies have shown that testicular oxidative stress, resulting from DM, is a major factor in male reproductive dysfunction [6, 65]. The Present study revealed significant decreases in antioxidant enzyme activity levels in the testes of diabetic rats, indicating the presence of oxidative stress [66]. The occurrence of oxidative stress in DM is attributed to the overproduction of superoxide, a free radical, induced by hyperglycemia and the mitochondrial electron-transport chain in various cells of the body. This superoxide can further get converted into more reactive species, leading to cellular damage [67]. The body’s normal response to this oxidative insult involves the deployment of antioxidant enzymes like CAT, UGT and GSH to neutralize these radicals [68].

The chemical components of EH-EtOH were directly responsible for the extract`potentiality to prevent and/or mitigate the testicular damage caused by STZ in diabetic rats. Metabolites profiling of *E*. *heterophylla* revealed for its richness in phenolics including phenolic acids, flavonoids, coumarins, and phloroglucinols, in addition to terpenes. These classes are well reported for their effective antioxidants and anti-inflammatory agents [69] and likely to have accounted for the observed protective effect against the testes damage in the diabetic model. Flavonoid and phenolic components found in most plant extracts exhibit potential antioxidant properties against oxidative stress and/or damage caused by neutralizing radicals [70, 71]. Mainly, the major phenolic acid derivatives detected in *E*. *heterophylla* were protocatechuic aldehyde, sinapoyl hexoside, feruloyl hexoside, and dimethoxy benzoic acid (P13,18, 20, and 27). Protocatechuic acid anti-inflammatory and antiapoptotic effects has been demonstrated in testicular tissue degeneration, [72]. Additionally, its aldehyde has shown antioxidant potential [73].

The observed decrease in antioxidant enzyme activity in diabetic group observed in the current study can account for hormonal disorders [25]. Hence, it is crucial to mitigate the production of ROS for effective treatment of reproductive damage in individuals with diabetes. Further, the role of inflammation in diabetic testicular complications is well understood, as the excessive expression of inflammatory cytokines can considerably hinder T4 production and impede the differentiation of spermatogonia, resulting in impaired gonadal functions, especially affecting Leydig cells’ ability to produce steroids [74].

In our study, elevated levels of various cytokines, including IL-1*β* and TNF-*α*, were detected in the testicular tissue of diabetic rats as a resultant of abovementioned oxidative stress. These cytokines contribute to inflammatory responses and also lead to a state of severe oxidative stress [75]. The involvement of pro-inflammatory mediators contributes to different types of reproductive dysfunction by triggering testicular damage, leading to significant loss of germ cells, testicular atrophy, and apoptosis. It has been demonstrated that there are interconnected relationships among inflammation, oxidative stress, apoptosis of sperm cells, and testicular injury [76, 77]. In diabetic rats treated with EH-EtOH, a pronounced antioxidant effect was observed as manifested by a significant increase in testicular levels of glutathione (GSH), catalase (CAT) and UGT activities, and in accordance with previous reports [13, 78]. Additionally, EH-EtOH demonstrated its anti-inflammatory properties, which were further supported by a notable decrease in the production of cytokines, including IL-6 and TNF-*α*. These results align with previous research studies [79, 80] in other species of *Euphorbia*.

The current study illuminates the anti-inflammatory action of EH-EtOH as its mechanistic action in mitigating destructive role of STZ on testicular tissues. The previous studies discovered that sinapic, ferulic acids, and dimethoxy benzoic acid derivatives possess anti-inflammatory, anti-apoptotic, and antioxidant activities. They reduced IL-1*β*, TNF-*α*, and IL6, decreased caspase-3 protein activity, retained the expression of Bcl-2 [81], activated Nrf2 [82], and repressed HMGB1/NF-κB axis [83].

Whereas the major flavonoids detected in *E*. *heterophylla* included isorhamnetin, quercetin, luteolin, apigenin, and diosmetin, and their glycoside derivatives *viz*. isovitexin-*C*-hexoside, quercetin-*O-*dihexoside, quercetin-*O*-hexoside, quercitrin, luteolin-*C*-hexoside, and isorhamnetin-*O*-hexoside (P21, 22, 26, 28, 29, 30, 34, 36, 37, and 40). They played a key role in the amelioration of STZ-induced oxidative stress and lipid peroxidation due to their significant anti-inflammatory and antioxidant potencies [69, 84, 85]. The presence of these compounds in *E*. *heterophylla* resulted in a decrease in pro-inflammatory cytokines and exhibited inhibitory effects on COX activity [86]. Interestingly, isovitexin and its glycosides were found to exert significant inhibitory action against TNF-*α* and interleukins, including IL-1*β* and IL-6 [87], in addition to the COX-2 and LOX-15 [88, 89]. Vitexin/ isovitexin and its derivatives are major C flavonoids in *E*. *heterophylla* are well reported for suppressing diabetic complications, such as adipose tissue disorders, impaired sexual and reproduction, pancreatic *β*-cell malfunction, hyperglycemia, diabetic neuropathy, liver disorders, diabetic nephropathy, vascular disease, platelet aggregation, and hypertension. These benefits were attributed to their antioxidant and anti-inflammatory abilities, and likewise inhibition of cell apoptosis and oxidative stress [90]. Studies have demonstrated that quercetin, luteolin, apigenin, isorhamnetin and their glycosides possessed antioxidant effects and elevated the production of endothelial NO synthase (eNOS), resulting in a substantial improvement in testicular structure and functions [91–93]. Aside from flavonoids, coumarins likewise detected in *E*. *heterophylla*, such as bicoumol (P 38), are reported for their anti-apoptotic, anti-androgenic, antioxidant, and anti-inflammatory capabilities mediating for preventing testicular dysfunction [94]. The antioxidant effects of coumarins are evident from their ability to decrease GSH and SOD, while simultaneously raising LH, lipid peroxides, and plasma total antioxidant capacity (TAC) [95]. Phlorolglucinols, a characteristic natural product class in *E*. *heterophylla* represented by isobutyrylphloroglucinol and dihydroxy methoxy methylacetophenone-*O*-hexoside (P15, 19), have been reported to increase sexual function and decrease serum glucose levels, hence improving the efficacy of sildenafil in treating sexual dysfunction in STZ-induced diabetic rats [96]. Such myriad of structures and effects present in *E*. *heterophylla* extract to prevent testicular injury in diabetic rats suggest for the existence of a synergistic action among EH-EtOH metabolites that has yet to be revealed by testing individual components.

A potential link between diabetes-induced testicular damage and VEGF lies in the dysregulation of VEGF expression and signaling in diabetic conditions [97]. Studies have shown that diabetes can negatively impact VEGF expression in the testes, and to further contribute to impaired angiogenesis and compromised vascular functions within the testes, as VEGF is a critical regulator of blood vessel growth and maintenance. Furthermore, reduction in VEGF expression in diabetic testes may also exacerbate oxidative stress and apoptosis. VEGF has been shown to possess antioxidant properties and can protect against oxidative stress-induced damage in various tissues, including the testes. Elshafey et al [98] demonstrated that the downregulation of VEGF signaling in rat testes resulted in increased oxidative stress and apoptosis, leading to testicular dysfunction. Therefore, decreased VEGF expression observed in diabetes-induced testicular damage could contribute to the oxidative stress and apoptotic processes that occur in diabetic testes. Our study revealed that EH-EtOH increased VEGF level concurrent with elevated levels of TNF-*α* and caspase-3 induced by STZ in testicular tissue. These findings provide additional evidence supporting the protective effects of EH-EtOH on reproductive system dysfunction and inflammation associated with diabetes.

The sufficient content of phenolic acids especially protocatechuic aldehyde, flavonoids, coumarins, and phloroglucinols, in addition to terpenes support our result in indirect regulating VEGF expression [99] but this role could be mainly owed to their antioxidant and anti-inflammatory activity.

In the current study, STZ-induced diabetic rats revealed an increase in serum E2 level with an obvious reduction in T4 level compared with control animals, and in accordance with previous results [100]. Changes in hormonal balance as manifested in the case of T4 and E2 could affect body homeostasis [101]. Thus, the high E2/T4 ratio in diabetic rats could be attributed to altered activity of aromatase, the enzyme responsible for converting androgen to E2 [102]. In addition, diabetes-induced testicular damage, characterized by oxidative stress, inflammation, and vascular dysfunction, is believed to contribute to the reduction in T4 levels. The impaired testicular functions associated with diabetes can disrupt Leydig cell activity, which is responsible for T4 production, leading to decreased T4 biosynthesis and secretion. In the current study, diabetic rats treated with dose-dependent EH-EtOH significantly led to a decrease in E2/T4 ratio as a positive effect likely mediated via the increase of catalase, GSH and UGT levels in testicular tissue indicating its beneficial effects on steroidogenesis and Leydig cell functions. The treatment with EH-EtOH rich in phenolics and flavonoids revealed the restoring the equilibrium hormonal status in diabetic rats as discussed by several studies but the definite mechanisms were illustrated by [103] where flavonoids did not only exert their antioxidant activity but also increased StAR expression in Leydig cells resulting in improvement of steroidogenesis thus improved gonadal functions.

The present study further assessed the expression of caspase-3, a key effector protein involved in apoptosis, a programmed cell death process that plays a significant role in testicular homeostasis and pathological status [104]. The results showed a notable increase in caspase-3 expression level inside the testicular tissue of diabetic rats. However, EH-EtOH diminished the apoptotic effect in testicular tissue caused by diabetes, indicative for the modulating consequence of EH-EtOH on caspase-3 apoptosis signaling pathway. These results are in accordance with Nna et al., 2020 [105] that revealed increased expression of caspase-3 inside tumor cells, indicating enhanced apoptotic activity. This suggests that caspase-3-mediated apoptosis may contribute to testicular damage associated with testicular germ cell tumors (TGCTs). Furthermore, other conditions that have led to testicular damage, such as testicular torsion or ischemia-reperfusion injury, have also been investigated using immunohistochemistry for caspase-3. A study by Shamsi-Gamchi and his co-workers [106] examined the effects of ischemia-reperfusion injury on testicular tissue in a rat model. Immunohistochemical staining for caspase-3 revealed increased expression in experimental group compared with the control group, indicating activation of apoptotic pathways and cell death as a consequence of testicular damage. Immunohistochemical analysis of caspase-3 provided valuable insights into the severity and progression of testicular damage and the extent and localization of the apoptotic activity in testicular tissue. Altogether, results of this study revealed the favored effect of EH-EtOH on testicular degeneration in STZ-induced diabetic rats. The effects of EH-EtOH on caspase-3 have been attributed to its anti-inflammatory and antioxidant properties. These effects are primarily mediated by phenolic acids (such as protocatechuic aldehyde, sinapoyl-*O*-hexoside, and feruloyl-*O*-hexoside), flavonoids, coumarins, and phloroglucinols, along with terpenes. Notably, these compounds do not exhibit direct activity on caspase-3 [107].

## 5. Conclusions

This aforementioned current study provides the first metabolite profile using UHPLC-ESI-Orbitrap-MS analysis and to report its potential to protect rats’ testicles from damage caused by STZ-induced DM. Several metabolites, including newly reported phytochemicals from *Euphorbia* plants such as isovitexin-*C*-hexoside, isorhamnetin-*O*-hexoside, diosmetin, and halfordin, were identified as part of this study. This study is the first to demonstrate the protective effect of EH-EtOH on testicular degeneration The potential protective effects of EH-EtOH were attributed to several mechanisms, including anti-inflammatory, antioxidant, anti-apoptotic actions, as well as hormonal mechanisms. These effects were mediated via induction of protein expression antioxidant levels including CAT, GSH and UGT, concurrent with decreased levels of inflammatory cytokines including PGE2, COX-2, IL-1*β* and TNF-*α*. These findings suggest that EH-EtOH holds promise as a beneficial therapeutic agent in protecting against testicular damage-induced by STZ-induced DM and has yet to be tested in other testicular dysfunction models. Additionally, future approaches were recommended to identify the best extraction solvents targeting the recovery and yield of these phytochemicals. Although the present findings presented a new strategy for using of the EH-EtOH against the testicular degeneration, but the limitations are the way of using of this extract in the human. Therefore, more advanced pre-clinical studies must be performed.

## Supporting information

S1 Fig MS/MS spectrum of caffeoyl-*O*-hexoside (P7) in negative ion mode. (DOCX)

S2 Fig MS/MS spectrum of protocatechuic acid (P11) in negative ion mode. (DOCX)

S3 Fig MS/MS spectrum of isovitexin-*C*-hexoside (P21) in negative ion mode. (DOCX)

S4 Fig MS/MS spectrum of quercetin-*O*-dihexoside (P22) in negative ion mode. (DOCX)

S5 Fig MS/MS spectrum of quercetin -*O*-hexoside (P26) in negative ion mode. (DOCX)

S6 Fig MS/MS spectrum of bicoumol (P38) in positive ion mode. (DOCX)

S7 Fig MS/MS spectrum of isobutyrylphloroglucinol (P15) in positive ion mode. (DOCX)
