# Correction: Protective effects of *Euphorbia heterophylla* against testicular degeneration in streptozotocin-induced diabetic rats in relation to phytochemical profile

**DOI:** 10.1371/journal.pone.0321595

**Published:** 2025-04-01

**Authors:** Ahmed M. Nagy, Heba A. Fahmy, Mohamed F. Abdel-Hameed, Rehab F. Taher, Alaa M. Ali, Mohamed M. Amin, Sherif M. Afifi, Tuba Esatbeyoglu, Mohamed A. Farag, Abdelsamed I. Elshamy

There is an error in affiliation 3 for authors Mohamed F. Abdel-Hameed and Mohamed M. Amin. The correct affiliation 3 is: Department of Pharmacology, National Research Centre, Giza, Egypt.
