# Supplementary material for: Protective effects of Euphorbia heterophylla against testicular degeneration in streptozotocin-induced diabetic rats in relation to phytochemical profile
Source: PLoS One. 2025 Jan 16;20(1):e0314781. doi: 10.1371/journal.pone.0314781 (PMC11737740; doi:10.1371/journal.pone.0314781)
Supplement: S1 Fig — (DOCX) [file pone.0314781.s001.docx]

**Supporting data**

**Protective effects of *Euphorbia heterophylla*** **against testicular degeneration in streptozotocin-induced diabetic rats in relation to phytochemical profile**

Ahmed M. Nagy^1^, [Heba A. Fahmy](https://pubs.rsc.org/en/results?searchtext=Author%3AHeba%20Fahmy)^2^, Mohamed F. Abdel-Hameed^3^, [Rehab F. Taher](https://sciprofiles.com/profile/1250825?utm_source=mdpi.com&utm_medium=website&utm_campaign=avatar_name)^4^, Alaa M. Ali^5^, Mohamed M. Amin^3^, Sherif M. Afifi^6^, Tuba Esatbeyoglu^7,*^, [Mohamed A. Farag](https://pubs.rsc.org/en/results?searchtext=Author%3AMohamed%20A.%20Farag)^8^, Abdelsamed I. Elshamy^4,^*

^1^ Department of Animal Reproduction &AI, National Research Center, 33 El Bohouth St., Dokki, Giza 12622, Egypt.

^2^ Pharmacognosy Department, Faculty of Pharmacy, Modern University for Technology & Information, Cairo, Egypt

^3^ Department of Pharmacology, Research Centre, 33 El Bohouth St., Dokki, Giza 12622, Egypt.

^4^ Department of Natural Compounds Chemistry, National Research Centre, 33 El Bohouth St., Dokki, Giza 12622, Egypt

^5^ Department of Pathology, Faculty of Veterinary Medicine, Cairo University, Giza 12211, Egypt;

^6^ Department for Life Quality Studies, Rimini Campus, University of Bologna, Corso d’Augusto 237, 47921 Rimini, Italy

^7^ Department of Molecular Food Chemistry and Food Development, Institute of Food and One Health, Gottfried Wilhelm Leibniz University Hannover, Am Kleinen Felde 30, 30167 Hannover, Germany

^8^ Pharmacognosy Department, Faculty of Pharmacy, Cairo University, Cairo, 11562, Egypt

Correspondence: [esatbeyoglu@lw.uni-hannover.de](mailto:esatbeyoglu@lw.uni-hannover.de) (T.E.); [elshamynrc@yahoo.com](mailto:elshamynrc@yahoo.com) (A.I.E.)


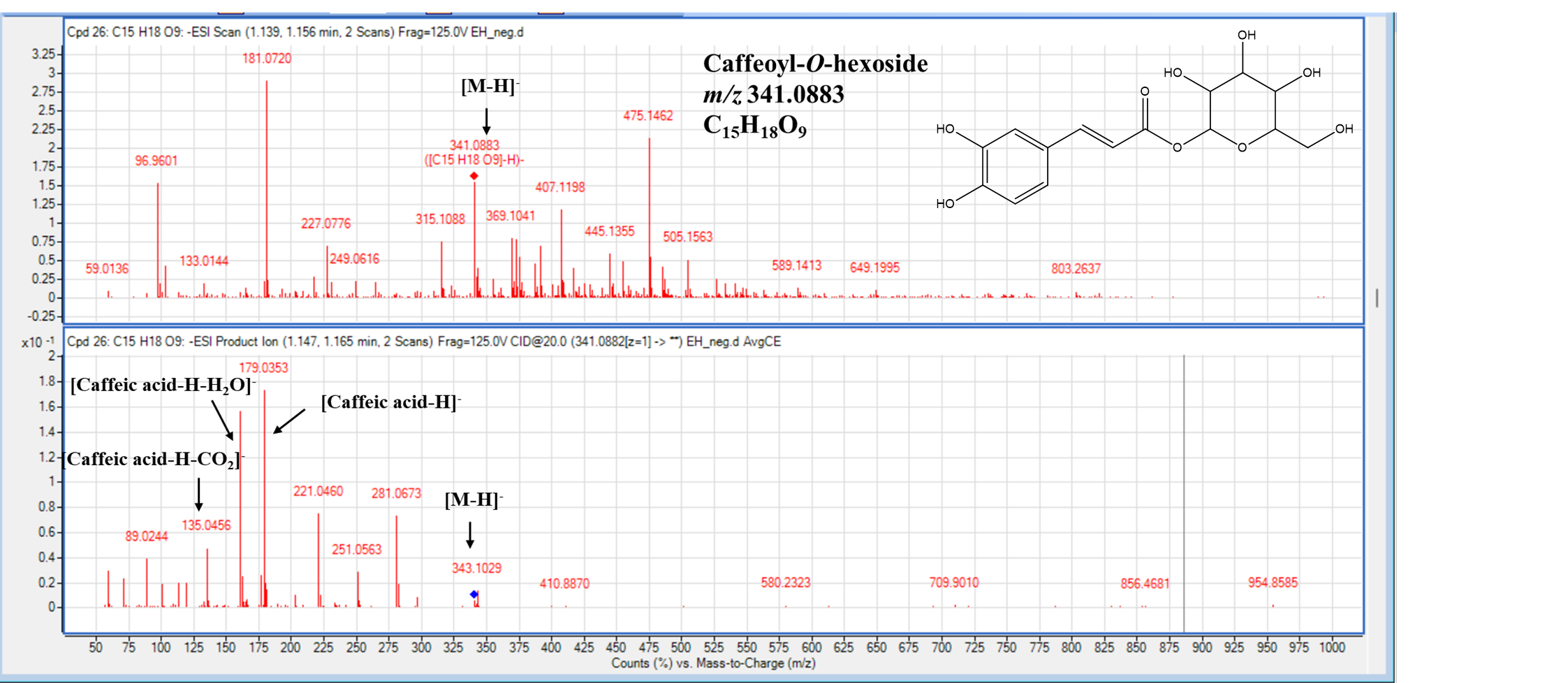


**Figure S1:** **MS/MS spectrum of Caffeoyl-*O*-hexoside (P7) in negative ion mode**
